# A 5700 year-old human genome and oral microbiome from chewed birch pitch

**DOI:** 10.1038/s41467-019-13549-9

**Published:** 2019-12-17

**Authors:** Theis Z. T. Jensen, Jonas Niemann, Katrine Højholt Iversen, Anna K. Fotakis, Shyam Gopalakrishnan, Åshild J. Vågene, Mikkel Winther Pedersen, Mikkel-Holger S. Sinding, Martin R. Ellegaard, Morten E. Allentoft, Liam T. Lanigan, Alberto J. Taurozzi, Sofie Holtsmark Nielsen, Michael W. Dee, Martin N. Mortensen, Mads C. Christensen, Søren A. Sørensen, Matthew J. Collins, M. Thomas P. Gilbert, Martin Sikora, Simon Rasmussen, Hannes Schroeder

**Affiliations:** 1 The Globe Institute, Faculty of Health and Medical Sciences, University of Copenhagen, Copenhagen, 1353 Denmark; 2 BioArch, Department of Archaeology, University of York, York, YO10 5DD UK; 3 Department of Bio and Health Informatics, Technical University of Denmark, Kongens, Lyngby 2800 Denmark; 4 Novo Nordisk Foundation Center for Protein Research, Faculty of Health and Medical Sciences, University of Copenhagen, Copenhagen, 2200 Denmark; 5 Centre for Isotope Research, University of Groningen, Groningen, 9747 AG The Netherlands; 6 The National Museum of Denmark, I.C. Modewegs Vej, Brede, Kongens Lyngby, 2800 Denmark; 7 Museum Lolland-Falster, Frisegade 40, Nykøbing Falster, 4800 Denmark; 8 McDonald Institute for Archaeological Research, University of Cambridge, Cambridge, CB2 3ER UK; 9 University Museum, NTNU, 7012 Trondheim, Norway

**Keywords:** Metagenomics, Population genetics, DNA sequencing, Archaeology

## Abstract

The rise of ancient genomics has revolutionised our understanding of human prehistory but this work depends on the availability of suitable samples. Here we present a complete ancient human genome and oral microbiome sequenced from a 5700 year-old piece of chewed birch pitch from Denmark. We sequence the human genome to an average depth of 2.3× and find that the individual who chewed the pitch was female and that she was genetically more closely related to western hunter-gatherers from mainland Europe than hunter-gatherers from central Scandinavia. We also find that she likely had dark skin, dark brown hair and blue eyes. In addition, we identify DNA fragments from several bacterial and viral taxa, including Epstein-Barr virus, as well as animal and plant DNA, which may have derived from a recent meal. The results highlight the potential of chewed birch pitch as a source of ancient DNA.

## Abstract (web summary)

Birch pitch is thought to have been used in prehistoric times as hafting material or antiseptic and tooth imprints suggest that it was chewed. Here, the authors report a 5,700 year-old piece of chewed birch pitch from Denmark from which they successfully recovered a complete ancient human genome and oral microbiome DNA.

## Introduction

Birch pitch is a black-brown substance obtained by heating birch bark and has been used as an adhesive and hafting agent as far back as the Middle Pleistocene^1,2^. Small lumps of this organic material are commonly found on archaeological sites in Scandinavia and beyond, and while their use is still debated, they often show tooth imprints, indicating that they were chewed^3^. Freshly produced birch pitch hardens on cooling and it has been suggested that chewing was a means to make it pliable again before using it, e.g. for hafting composite stone tools. Medicinal uses have also been suggested, since one of the main constituents of birch pitch, betulin, has antiseptic properties^4^. This is supported by a large body of ethnographic evidence, which suggests that birch pitch was used as a natural antiseptic for preventing and treating dental ailments and other medical conditions^3^. The oldest examples of chewed pitch found in Europe date back to the Mesolithic period and chemical analysis by Gas Chromatography-Mass Spectrometry (GC-MS) has shown that many of them were made from birch (*Betula pendula*)^3^.

**Fig. 1:**
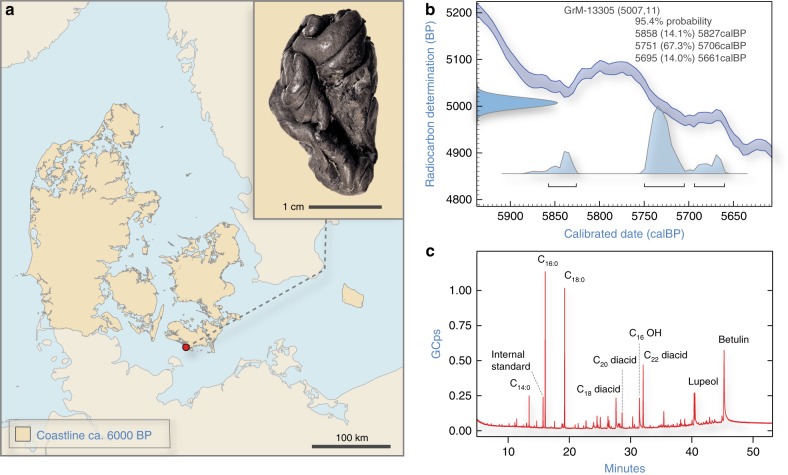
A chewed piece of birch pitch from southern Denmark. (**a**) Photograph of the Syltholm birch pitch and its find location at the site of Syltholm on the island of Lolland, Denmark (map created using data from Astrup^78^). (**b**) Calibrated date for the Syltholm birch pitch (5,858–5,661 cal. BP; 5,007 ± 7). (**c**) GC-MS chromatogram of the Syltholm pitch showing the presence of a series of dicarboxylic acids (Cxx diacid) and saturated fatty acids (Cxx:0) and methyl 16-Hydroxyhexadecanoate (C_16_OH) together with the triterpenes betulin and lupeol, which are characteristic of birch pitch^3^.

Recent work by Kashuba et al^5^. has shown that pieces of chewed birch pitch contain ancient human DNA, which can be used to link the material culture and genetics of ancient populations. In the current study, we analyse a further piece of chewed birch pitch, which was discovered at a Late Mesolithic/Early Neolithic site in southern Denmark (Fig. 1a; Supplementary Note 1) and demonstrate that it does not only contain ancient human DNA, but also microbial DNA that reflects the oral microbiome of the person who chewed the pitch, as well as plant and animal DNA which may have derived from a recent meal. The DNA is so exceptionally well preserved that we were able to recover a complete ancient human genome from the sample (sequenced to an average depth of coverage of 2.3×), which is particularly significant since, so far, no human remains have been recovered from the site^6^. The results highlight the potential of chewed birch pitch as a source of ancient human and non-human DNA, which can be used to shed light on the population history, health status, and even subsistence strategies of ancient populations.

## Results

### Radiocarbon dating and chemical analysis

Radiocarbon dating of the specimen yielded a direct date of 5,858–5,661 cal. BP (GrM-13305; 5,007 ± 11) (Fig. 1b; Supplementary Note 2), which places it at the onset of the Neolithic period in Denmark. Chemical analysis by Fourier-Transform Infrared (FTIR) spectroscopy produced a spectrum very similar to modern birch pitch (Supplementary Fig. 4) and GC-MS revealed the presence of the triterpenes betulin and lupeol, which are characteristic of birch pitch (Fig. 1c; Supplementary Note 3)^3^. The GC-MS spectrum also shows a range of dicarboxylic acids and saturated fatty acids, which are all considered intrinsic to birch pitch and thus support its identification^7^.

### DNA sequencing

We generated approximately 390 million DNA reads for the sample, nearly a third of which could be uniquely mapped to the human reference genome (hg19) (Supplementary Table 2). The human reads displayed all the features characteristic of ancient DNA, including (i) short average fragment lengths, (ii) an increased occurrence of purines before strand breaks, and (iii) an increased frequency of apparent cytosine (C) to thymine (T) substitutions at 5′-ends of DNA fragments (Supplementary Fig. 6) and the amount of modern human contamination was estimated to be around 1–3% (Supplementary Table 3). In addition to the human reads, we generated around 7.3 Gb of sequence data (68.8%) from the ancient pitch that did not align to the human reference genome.

### DNA preservation and genome reconstruction

With over 30%, the human endogenous DNA content in the sample was extremely high and comparable to that found in well-preserved teeth and petrous bones^8^. We used the human reads to reconstruct a complete ancient human genome, sequenced to an effective depth-of-coverage of 2.3×, as well as a high-coverage mitochondrial genome (91×), which was assigned to haplogroup K1e (see Methods). To further investigate the preservation of the human DNA in the sample we calculated a molecular decay rate (*k*, per site per year) and find that it is comparable to that of other ancient human genomes from temperate regions (Supplementary Table 3).

### Sex determination and phenotypic traits

Based on the ratio between high-quality reads (MAPQ ≥ 30) mapping to the X and Y chromosomes, respectively^9^, we determined the sex of the individual whose genome we recovered to be female. To predict her hair, eye and skin colour we imputed genotypes for 41 SNPs (Supplementary Data 1) included in the HIrisPlex-S system^10^ and find that she likely had dark skin, dark brown hair, and blue eyes (Supplementary Data 2). We also examined the allelic state of two SNPs linked with the primary haplotype associated with lactase persistence in humans and found that she carried the ancestral allele for both (Supplementary Data 1), indicating that she was lactase non-persistent.

### Genetic affinities

**Fig. 2:**
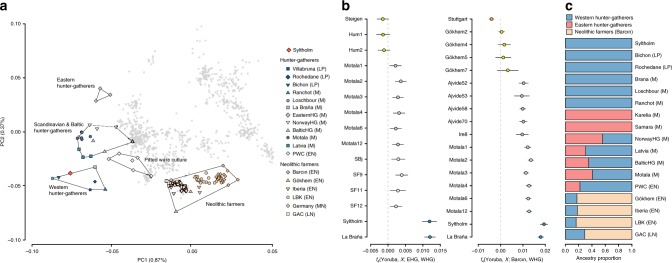
Genetic affinities of the Syltholm individual. **a** Principal component analysis of modern Eurasian individuals (in grey) and a selection of over 100 previously published ancient genomes, including the Syltholm genome. The ancient individuals were projected on the modern variation (see Methods). **b** Allele-sharing estimates between the Syltholm individual, other Mesolithic and Neolithic individuals, and WHGs versus EHGs and Neolithic farmers, respectively, as measured by the statistic *f*_4_(Yoruba, *X*; EHG/Barcın, WHG). **c** Ancestry proportions based on *qpAdm*^12^, specifying WHG, EHG, and Neolithic farmers (Barcın) as potential ancestral source populations. *PWC* Pitted Ware Culture, *LBK* Linearbandkeramik, *GAC* Globular Amphora Culture, *LP* Late Paleolithic, *M* Mesolithic, *EN* Early Neolithic, *MN* Middle Neolithic, *LN* Late Neolithic. Data are shown in Supplementary Tables 4–6.

We called 593,102 single nucleotide polymorphisms (SNPs) in our ancient genome that had previously been genotyped in a dataset of >1000 present-day individuals from a diverse set of Eurasian populations^11^, as well as >100 previously published ancient genomes (Supplementary Data 3). Figure 2a shows a principal component analysis (PCA) where she clusters with western hunter-gatherers (WHGs). Allele-sharing estimates based on *f*_4_-statistics show the same overall affinity to WHGs (Fig. 2b). This is also reflected in the *qpAdm* analysis^12^ (see Methods) which demonstrates that a simple one way model assuming 100% WHG ancestry cannot be rejected in favour of more complex models (Fig. 2c; Supplementary Table 6). To formally test this result we computed two sets of *D*-statistics of the form *D*(Yoruba, EHG/Barcın; test, WHG) and find no evidence for significant levels of EHG or Neolithic farmer gene flow (Supplementary Fig. 7; Supplementary Tables 7, 8).

### Metataxonomic profiling of non-human reads

**Fig. 3:**
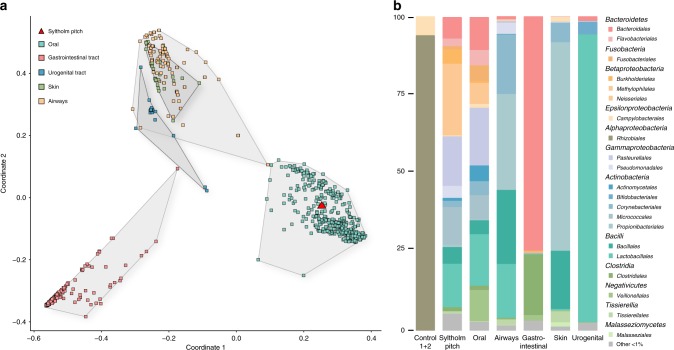
Metagenomic profile of the Syltholm birch pitch. **a** PCoA with Bray-Curtis at genera level with 689 microbiomes from HMP^14^ and the Syltholm sample (see Methods). **b** Order-level microbial composition of the Syltholm sample compared to a control sample (soil) and metagenome profiles of healthy human subjects at five major body sites from the HMP^14^, visualised using MEGAN6^15^.

To broadly characterise the taxonomic composition of the non-human reads in the sample, we used MetaPhlan2^13^, a tool specifically designed for the taxonomic profiling of short-read metagenomic shotgun data (see Methods; Supplementary Data 4). Figure 3a shows a principal coordinate analysis where we compare the microbial composition of our sample to that of 689 microbiome profiles from the Human Microbiome Project (HMP)^14^. We find that our sample clusters with modern oral microbiome samples in the HMP dataset. This is also reflected in Fig. 3b which shows the order-level microbial composition of our sample compared to two soil samples from the same site and metagenome profiles of healthy human subjects at five major body sites from the HMP^14^, visualised using MEGAN6^15^.

### Oral microbiome characterisation

To further characterise the microbial taxa present in the ancient pitch and to obtain species-specific assignments we used MALT^16^, a fast alignment and taxonomic binning tool for metagenomic data that aligns DNA sequencing reads to a user-specified database of reference sequences (see Methods; Supplementary Data 5). As expected, a large number of reads could be assigned to oral taxa, such as *Neisseria subflava* and *Rothia mucilaginosa*, as well as several bacteria included in the red complex (i.e. *Porphyromonas gingivalis*, *Tannerella forsythia*, and *Treponema denticola*) (see Table 1). In addition, we recovered 593 reads that were assigned to Epstein–Barr virus (Human gammaherpesvirus 4). We validated each taxon by examining the edit distances, coverage distributions, and post-mortem DNA damage patterns (see Supplementary Note 5).

**Table 1:** List of non-human taxa identified in the Syltholm pitch, including the 40 most abundant oral bacterial taxa, viruses, and eukaryotes. Bacteria in the red complex are denoted with an asterisk. Depth (DoC) and breadth of coverage (>1x) were calculated using BEDTools^72^. Deamination rates at the 5’ ends of DNA fragments were estimated using mapDamage 2.0.9^59^. -Δ% refers to the negative difference proportion introduced by Hübler *et al*^79^. (see Supplementary Note 5).

Species	Reads	Fragment length (bp)	DoC	SD DoC	>1x (%)	C-T 5′(%)	−Δ%
Bacteria							
*Neisseria subflava*	308,732	56	7.5	6.2	83.7	14.5	0.9
*Rothia mucilaginosa*	296,610	52	6.9	5.6	82.3	14.0	0.9
*Streptococcus pneumoniae*	176,782	57	4.7	6.3	65.7	13.8	0.9
*Neisseria cinerea*	153,683	58	4.9	5.1	71.7	15.1	1.0
*Lautropia mirabilis*	117,040	53	2.0	1.9	71.9	13.0	1.0
*Neisseria meningitidis*	100,540	51	2.3	4.3	42.4	14.9	0.9
*Aggregatibacter segnis*	95,670	58	2.8	2.8	73.3	14.5	0.9
*Neisseria elongata*	68,407	54	1.6	1.9	67.6	15.1	0.9
*Prevotella intermedia*	65,324	56	1.2	1.4	55.0	16.2	0.9
*Streptococcus* sp. ChDC B345	52,614	61	1.6	2.7	50.3	13.8	0.9
*Streptococcus* sp. 431	43,787	59	1.2	1.9	47.5	13.6	0.8
*Aggregatibacter aphrophilus*	43,231	56	1.1	1.6	50.4	15.0	0.8
*Streptococcus pseudopneumoniae*	38,832	61	1.1	2.4	34.9	14.4	0.9
*Capnocytophaga leadbetteri*	36,461	59	0.9	1.1	49.8	14.0	0.8
*Corynebacterium matruchotii*	36,070	52	0.7	0.9	44.0	13.0	1.0
*Gemella morbillorum*	32,284	63	1.2	1.5	56.4	16.3	1.0
*Streptococcus viridans*	27,840	60	0.8	1.5	36.5	14.5	1.0
*Neisseria gonorrhoeae*	27,704	53	0.7	2.0	21.3	15.0	1.0
*Neisseria sicca*	27,290	57	0.6	1.4	22.5	13.7	0.9
*Fusobacterium nucleatum*	26,783	64	0.8	1.1	47.8	14.1	0.9
*Prevotella fusca*	26,295	57	0.5	0.7	34.6	15.7	1.0
*Kingella kingae*	25,811	55	0.7	1.0	44.2	14.4	1.0
*Ottowia* sp. 894	25,425	52	0.5	0.7	34.6	14.4	1.0
*Streptococcus* sp. NPS 308	24,937	59	0.8	1.4	37.5	14.3	0.8
*Actinomyces oris*	24,029	52	0.4	0.7	29.8	12.7	1.0
*Streptococcus australis*	23,777	60	0.7	1.3	31.5	13.8	1.0
*P. propionicum*	22,864	50	0.3	0.6	26.8	13.2	0.9
*Haemophilus* sp. 036	19,707	62	0.7	1.5	28.4	14.5	1.0
Porphyromonas gingivalis*	17,651	55	0.4	0.7	32.2	17.2	1.0
*Capnocytophaga gingivalis*	16,734	58	0.3	0.6	27.1	15.0	1.0
*Neisseria polysaccharea*	14,442	57	0.4	1.4	15.0	15.8	1.0
Tannerella forsythia*	14,187	55	0.2	0.5	19.8	15.3	1.0
*Streptococcus* sp. A12	13,232	59	0.4	0.9	24.9	14.6	0.9
*Capnocytophaga sputigena*	12,587	58	0.2	0.5	19.9	14.7	0.9
*Neisseria lactamica*	11,971	56	0.3	1.0	14.2	14.2	0.8
Treponema denticola*	11,379	59	0.2	0.5	19.5	14.0	0.8
*Rothia dentocariosa*	10,944	54	0.2	0.5	20.0	13.6	1.0
*Tannerella* sp. HOT-286	10,397	53	0.2	0.5	15.7	14.0	1.0
*Actinomyces meyeri*	10,105	51	0.3	0.5	21.3	14.0	1.0
*Filifactor alocis*	9,948	61	0.3	0.6	25.6	15.0	1.0
Viruses							
*Epstein-Barr virus*	593	51	0.2	0.4	13.3	17.8	1.0
Eukaryotes							
*Anas platyrhynchos*	55,986	51	<0.1	0.05	0.2	15.6	1.0
*Corylus avellana*	8,615	55	<0.1	0.04	0.1	19.7	1.0
*Betula pendula*	3,291	54	<0.1	0.02	<0.1	16.1	1.0

### Pneumococcal DNA

**Fig. 4:**
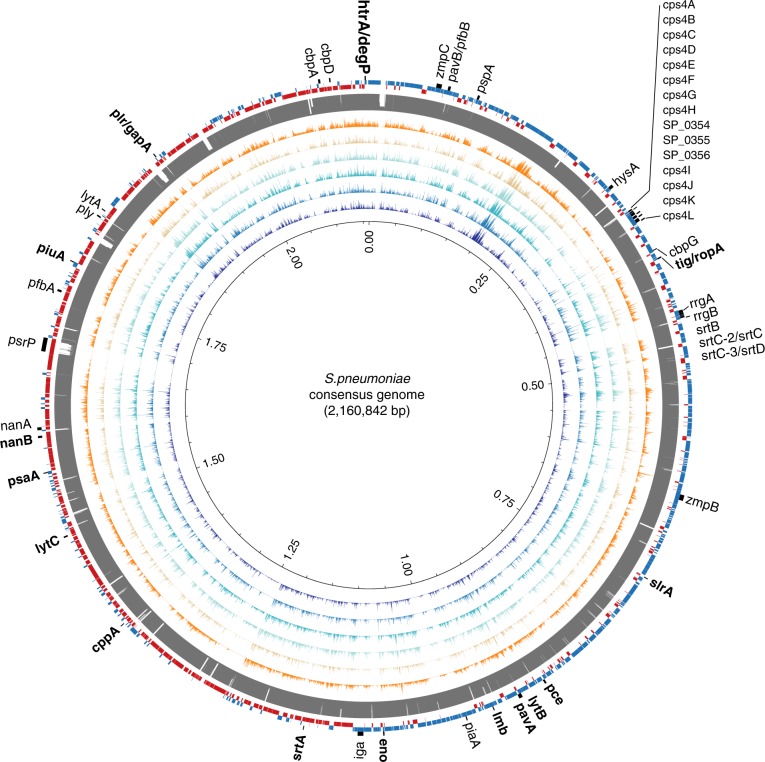
*Streptococcus pneumoniae* consensus genome reconstructed from metagenomic sequences recovered from the ancient pitch. From outer to inner ring: *S. pneumoniae* virulence genes (black, shared genes are shown in bold); *S. pneumoniae* coding regions on the positive (blue) and negative (red) strand; mappability (grey); sequence depth for the Syltholm pitch (orange), HOMP sample SRS014468 (light brown), SRS019120 (light blue), SRS013942 (turquoise), SRS015055 (blue), and SRS014692 (dark blue). Sequence depths were calculated by aligning to the *S. pneumoniae* TIGR4 reference genome and visualised in 100 bp windows using Circos^73^.

We also identified several species belonging to the Mitis group of streptococci (Table 1), including *Streptococcus viridans* and *Streptococcus pneumoniae*. We reconstructed a consensus genome from the *S. pneumoniae* reads (Fig. 4) and estimated the number of heterozygous sites (2,597) (see Methods) which indicates the presence of multiple strains. To assess the virulence of the *S. pneumoniae* strains recovered from the ancient pitch, we aligned the contigs against the full Virulence Factor Database^17^ in order to identify known *S. pneumoniae* virulence genes (see Methods). We identified 26 *S. pneumoniae* virulence factors within the ancient sample, including capsular polysaccharides (CPS), streptococcal enolase (Eno), and pneumococcal surface antigen A (PsaA) (see Supplementary Data 6).

### Plant and animal DNA

Lastly, we used a taxonomic binning pipeline specifically designed for ancient environmental DNA^18^ to taxonomically classify the non-human reads in the sample that mapped to other Metazoa (animals) and Viridiplantae (plants). We only parsed taxa with classified reads accounting for >1% of all reads in each of the two kingdoms and a declining edit distance distribution after edit distance 0 (Supplementary Data 7). We then validated each identified taxon as described above (see Supplementary Note 5). Using these criteria, we identified DNA from two plant species in the ancient sample, including birch (*Betula pendula*) and hazelnut (*Corylus avellana*). In addition, we detected over 50,000 reads that were assigned to mallard (*Anas platyrhynchos*).

## Discussion

We successfully extracted and sequenced ancient DNA from a 5700-year-old piece of chewed birch pitch from southern Denmark. In addition to a complete ancient human genome (2.3×) and mitogenome (91×), we recovered plant and animal DNA, as well as microbial DNA from several oral taxa. Analysis of the human reads revealed that the individual whose genome we recovered was female and that she likely had dark skin, dark brown hair and blue eyes. This combination of physical traits has been previously noted in other European hunter-gatherers^19–22^, suggesting that this phenotype was widespread in Mesolithic Europe and that the adaptive spread of light skin pigmentation in European populations only occurred later in prehistory^23^. We also find that she had the alleles associated with lactase non-persistence, which fits with the notion that lactase persistence in adults only evolved fairly recently in Europe, after the introduction of dairy farming with the Neolithic revolution^24,25^.

From a population genetics point of view, the human genome also offers fresh insights into the early peopling of southern Scandinavia. Recent studies of ancient hunter-gatherer genomes from Sweden and Norway^23^ have shown that, following the retreat of the ice sheets around 12–11 ka years ago, Scandinavia was colonised by two separate routes, one from the south (presumably via Denmark) and one from the northeast, along the coast of present-day Norway. This is supported by the fact that hunter-gatherers from central Scandinavia carry different levels of WHG and EHG ancestry, which reached central Scandinavia from the south and northeast, respectively^23^. Although we only analysed a single genome, the fact that the Syltholm individual does not carry any EHG ancestry confirms this scenario and suggests that EHGs did not reach southern Denmark at this point in prehistory.

The Syltholm genome (5700 years cal. BP) dates to the period immediately following the Mesolithic-Neolithic transition in Denmark. Culturally, this period is marked by the transition from the Late Mesolithic Ertebølle culture (c. 7300–5900 cal. BP) with its flaked stone artefacts and typical T-shaped antler axes, to the early Neolithic Funnel Beaker culture (c. 5900–5300 cal. BP) with its characteristic pottery, polished flint artefacts, and domesticated plants and animals^26^. In Denmark, the transition from hunting and gathering to farming has often been described as a relatively rapid process, with dramatic shifts in settlement patterns and subsistence strategies^27^. However, it is still unclear to what extent this transition was driven by the arrival of farming communities as opposed to the local adaptation of farming practices by resident hunter-gatherer populations.

Our analyses have shown that the Syltholm individual does not carry any Neolithic farmer ancestry, suggesting that the genetic impact of Neolithic farming communities in southern Scandinavia might not have been as instant or pervasive as once thought^28^. While the mtDNA we recovered belongs to haplogroup K1e, which is more commonly associated with early farming communities^29–31^, there is mounting evidence to suggest that this lineage was already present in Mesolithic Europe^32–34^. Overall, the lack of Neolithic farmer ancestry is consistent with evidence from elsewhere in Europe, which suggests that genetically distinct hunter-gatherer groups survived for much longer than previously assumed^35–37^. These WHG “survivors” might have triggered the resurgence of hunter-gatherer ancestry that is proposed to have occurred in central Europe between 7000 and 5000 BP^12^.

In addition to the human data, we recovered ancient microbial DNA from the pitch which could be shown to have a human oral microbiome signature. Previous studies^38–40^ have demonstrated that calcified dental plaque (dental calculus) provides a robust biomolecular reservoir that allows direct and detailed investigations of ancient oral microbiomes. However, unlike dental calculus, which represents a long-term reservoir of the oral microbiome built up over many years, the microbiota found in ancient mastics are more likely to give a snapshot of the species active at the time. As such, they provide a useful source of information regarding the evolution of the human oral microbiome that can complement studies of ancient dental calculus.

The majority of the bacterial taxa we identified (Table 1) are classified as non-pathogenic, commensal species that are considered to be part of the normal microflora of the human mouth and the upper respiratory tract, but may become pathogenic under certain conditions. In addition, we identified three species (*Porphyromonas gingivalis*, *Tannerella forsythia*, and *Treponema denticola*) included in the so-called red complex, a group of bacteria that are categorised together based on their association with severe forms of periodontal disease^41^. Furthermore, we identified several thousand reads that could be assigned to different bacterial species in the Mitis group of streptococci, including *Streptococcus pneumoniae*, a major human pathogen that is responsible for the majority of community-acquired pneumonia which still causes around 1–2 million infant deaths worldwide, every year^42^.

*S. pneumoniae* has a remarkable capacity to remodel its genome through the uptake of exogenous DNA from other pneumococci and closely related oral streptococci^42^. Understanding this process and the distribution of pneumococcal virulence factors between different strains can help our understanding of *S. pneumoniae* pathogenesis. We identified 26 *S. pneumoniae* virulence factors within our ancient sample, including several that are involved in host colonisation (e.g. adherence to host cells and tissues, endocytosis) and the evasion and subversion of the host’s immune response (Supplementary Data 6). While more research is needed to fully understand the evolution of this important human pathogen and its ability to cause disease, our capacity to recover virulence factors from ancient samples opens up promising avenues for future research.

In addition to the bacterial taxa, we identified 593 reads that could be assigned to the Epstein–Barr virus (EBV). Previous studies^43,44^ have demonstrated the great potential of ancient DNA for studying the long-term evolution of blood borne viruses. Formally known as Human gammaherpesvirus 4, EBV is one of the most common human viruses infecting over 90% of the world’s adult population^45^. Most EBV infections occur during childhood and in the vast majority of cases they are asymptomatic or they carry symptoms that are indistinguishable from other mild, childhood diseases. However, in some cases EBV can cause infectious mononucleosis (glandular fever)^46^ and it has also been associated with various lymphoproliferative diseases, such as Hodgkin's lymphoma and hemophagocytic lymphohistiocytosis, as well as higher risks of developing certain autoimmune diseases, such as dermatomyositis and multiple sclerosis^47,48^.

Lastly, we identified several thousand reads that could be confidently assigned to different plant and animal species, including birch (*B. pendula*), hazelnut (*C. avellana*), and mallard (*A. platyrhynchos*). While the presence of birch DNA is easily explained as it is the source of the pitch, we propose that the hazelnut and mallard DNA may derive from a recent meal. This is supported by the faunal evidence from the site, which is dominated by wild taxa, including *Anas* sp. and hazelnuts^6,49^. In addition, there is evidence from many other Mesolithic and Early Neolithic sites in Scandinavia for hazelnuts being gathered in large quantities for consumption^50^. Together with the faunal evidence, the ancient DNA results support the notion that the people at Syltholm continued to exploit wild resources well into the Neolithic and highlight the potential of ancient DNA analyses of chewed pieces of birch pitch for palaeodietary studies.

In summary, we have shown that pieces of chewed birch pitch are an excellent source of ancient human and non-human DNA. In the process of chewing, the DNA becomes trapped in the pitch where it is preserved due to the aseptic and hydrophobic properties of the pitch which both inhibits microbial and chemical decay. The genomic information preserved in chewed pieces of birch pitch offers a snapshot of people's lives, providing information on genetic ancestry, phenotype, health status, and even subsistence. In addition, the microbial DNA provides information on the composition of our ancestral oral microbiome and the evolution of specific oral microbes and important human pathogens.

## Methods

### Sample preparation and DNA extraction

We sampled c. 250 mg from the specimen for DNA analysis. Briefly, the sample was washed in 5% bleach solution to remove any surface contamination, rinsed in molecular biology grade water and left to dry. We tested three different extraction methods using between 20–50 mg of starting material: For method (1), 1 ml of lysis buffer containing 0.45 M EDTA (pH 8.0) and 0.25 mg/ml Proteinase K was added to the sample and left to incubate on a rotor at 56 °C. After 12 h the supernatant was removed and concentrated down to ~150 µl using Amicon Ultra centrifugal filters (MWCO 30 kDa), mixed 1:10 with a PB-based binding buffer^51^, and purified using MinElute columns, eluting in 30 µl EB. For method (2) the sample was digested and purified as above, but with the addition of a phenol-chloroform clean-up step. Briefly, 1 ml phenol (pH 8.0) was added to the lysis mix, followed by 1 ml chloroform:isoamyl alcohol. The supernatant was concentrated and purified, as described above. For method (3) the sample was dissolved in 1 ml chloroform:isoamylalcohol. The dissolved sample was then resuspended in 1 ml molecular grade water and purified as described above. DNA extracts prepared using a Proteinase K-based lysis buffer followed by a phenol-chloroform based purification step produced the best results in terms of the endogenous human DNA content (see Supplementary Table 1); however, following metagenomic profiling the extracts were found to be contaminated with *Delftia* spp., a known laboratory contaminant^52^. The contaminated libraries were excluded from metagenomic profiling.

### Negative controls

We included no template controls (NTC) during the DNA extraction and library preparation steps. The NTCs prepared with the additional phenol-chloroform step were also found to be contaminated with *Delftia* spp., suggesting that the contaminants were introduced during this step. In addition, we included two soil samples from the site, weighing c. 2 g each, as negative controls. DNA was extracted as described above using 3 ml EDTA-based lysis buffer followed by 9 ml 25:24:1 phenol:chloroform:isoamyl alcohol mixture to account for the larger amount of starting material. The sequencing results are reported in Supplementary Table 1.

### Library preparation and sequencing

16 µl of each DNA extract were built into double-stranded libraries using a recently published protocol that was specifically designed for ancient DNA^53^. One extraction NTC was included, as well as a single library NTC. 10 µl of each library were amplified in 50 µl reactions for between 15 and 28 cycles, using a dual indexing approach^54^. The optimal number of PCR cycles was determined by qPCR (MxPro 3000, Agilent Technologies). The amplified libraries were purified using SPRI-beads and quantified on a 2200 TapeStation (Agilent Technologies) using High Sensitivity tapes. The amplified and indexed libraries were then pooled in equimolar amounts and sequenced on 1/8 of a lane of an Illumina HiSeq 2500 run in SR mode. Following initial screening, additional reads were obtained by pooling libraries #2, #3, and #4 in molar fractions of 0.2, 0.4, and 0.4, respectively and sequencing them on one full lane of an Illumina HiSeq 2500 run in SR mode.

### Data processing

Base calling was performed using Illumina’s bcl2fastq2 conversion software v2.20.0. Only sequences with correct indexes were retained. FastQ files were processed using PALEOMIX v1.2.12^55^. Adapters and low quality reads (Q < 20) were removed using AdapterRemoval v2.2.0^56^, only retaining reads >25 bp. Trimmed and filtered reads were then mapped to hg19 (build 37.1) using BWA^57^ with seed disabled to allow for better sensitivity^58^, as well as filtering out unmapped reads. Only reads with a mapping quality ≥30 were kept and PCR duplicates were removed. MapDamage 2.0.9^59^ was used to evaluate the authenticity of the retained reads as part of the PALEOMIX pipeline^55^, using a subsample of 100k reads per sample (Supplementary Fig. 6). For the population genomic analyses, we merged the ancient sample with individuals from the Human Origin dataset^11^ and >100 previously published ancient genomes (Supplementary Data 1). At each SNP in the Human Origin dataset, we sampled the allele with more reads in the ancient sample, resolving ties randomly, resulting in a pseudohaploid ancient sample.

### MtDNA analysis and contamination estimates

We used Schmutzi^60^ to determine the endogenous consensus mtDNA sequence and to estimate present-day human contamination. Reads were mapped to the Cambridge reference sequence (rCRS) and filtered for MAPQ ≥ 30. Haploid variants were called using the *endoCaller* program implemented in Schmutzi^60^ and only variants with a posterior probability exceeding 50 on the PHRED scale (probability of error: 1/100,000) were retained. We then used Haplogrep v2.2^61^ to determine the mtDNA haplogroup, specifying PhyloTree (build 17) as the reference phylogeny^62^. Contamination estimates were obtained using Schmutzi’s *mtCont* program and a database of putative modern contaminant mitochondrial DNA sequences.

### Genotype imputation

We used ANGSD^63^ to compute genotype likelihoods in 5 Mb windows around 43 SNPs associated with skin, eye, and hair colour^10^ and lactase persistence into adulthood (Supplementary Data 2). Missing genotypes were imputed using impute2^64^ and the pre-phased 1000 Genome reference panel^65^, provided as part of the impute2 reference datasets. We used multiple posterior probability thresholds, ranging from 0.95 to 0.50, to filter the imputed genotypes. The imputed genotypes were uploaded to the HIrisPlex-S website^10^ to obtain the predicted outcomes for the pigmentation phenotypes (Supplementary Data 3).

### Principal component analysis

Principal component analysis was performed using smartPCA^66^ by projecting the ancient individuals onto a reference panel including >1000 present-day Eurasian individuals from the HO dataset^11^ using the option lsq project. Prior to performing the PCA the data set was filtered for a minimum allele frequency of at least 5% and a missingness per marker of at most 50%. To mitigate the effect of linkage disequilibrium, the data were pruned in a 50-SNP sliding window, advanced by 10 SNPs, and removing sites with an R^2^ larger than 0, resulting in a final data set of 593,102 SNPs.

### *D*- and *f*-statistics

*D*- and *f*-statistics were computed using *AdmixTools*^67^. To estimate the amount of shared drift between the Syltholm genome and WHG versus EHG and Neolithic farmers, respectively, we computed two sets of *f*_4_-statistics of the form *f*_4_(Yoruba, *X*; EHG/Barcın, WHG) where “*X*” stands for the test sample. Standard errors were calculated using a weighted block jackknife. To confirm the absence of EHG and Neolithic farmer gene flow in the Syltholm genome and to contrast this result with those obtained for other Mesolithic and Neolithic individuals from Scandinavia, we computed two sets of *D*-statistics of the form *D*(Yoruba, EHG/Barcın; *X*, WHG) testing whether “*X*” forms a clade to the exclusion of EHG and Neolithic farmers (represented by Barcın), respectively.

### qpAdm

Admixture proportions were modeled using *qpAdm*^12^, specifying Mesolithic Western European hunter-gatherers (WHG), Eastern hunter-gatherers (EHG) and early Neolithic Anatolian farmers (Barcın), as possible ancestral source populations. We present the model with the lowest number of source populations that fits the data, as well as the model with all three admixture components (see Supplementary Table 6). When estimating the admixture proportions for WHGs and EHGs, the test sample was excluded from their respective reference populations.

### MetaPhlan

We used MetaPhlan2^13^ to create a metagenomic profile based on the non-human reads (Supplementary Data 4). The reads were first aligned to the MetaPhlan2 database^13^ using Bowtie2 v2.2.9 aligner^68^. PCR duplicates were removed using PALEOMIX filteruniquebam^58^. For cross-tissue comparisons 689 human microbiome profiles published in the Human Microbiome Project Consortium^14^ were initially used, comprising samples from the mouth (*N* = 382), skin (*N* = 26), gastrointestinal tract (*N* = 138), urogenital tract (*N* = 56), airways and nose (*N* = 87). The oral HMP samples consist of attached/keratinised gingiva (*N* = 6), buccal mucosa (*N* = 107), palatine tonsils (*N* = 6), tongue dorsum (*N* = 128), throat (*N* = 7), supragingival plaque (*N* = 118), and subgingival plaque (*N* = 7). Pairwise ecological distances among the profiles were computed at genus and species level using taxon relative abundances and the vegdist function from the vegan package in R^69^. These were used for principal coordinate analysis (PCoA) of Bray–Curtis distances in R using the pcoa function included in the APE package^70^. Subsequently, we calculated the average relative abundance of each genus for each of the body sites present in the Human Microbiome Project and visualised the abundance of microbial orders of our sample and the HMP body sites with MEGAN6^15^.

### MALT

To further characterise the metagenomic reads we performed microbial species identification using MALT v. 0.4.1 (Megan ALignment Tool)^16^, a rapid sequence-alignment tool specifically designed for the analysis of metagenomic data. All complete bacterial (*n* = 12,426) and viral (*n* = 8094) genomes were downloaded from NCBI RefSeq on 13 November 2018, and all complete archaeal (*n* = 280) genomes were downloaded from NCBI RefSeq on 17 November 2018 to create a custom database. In an effort to exclude genomes that may consist of composite sequences from multiple organisms, the following entries were excluded:

GCF_000922395.1 uncultured crAssphage

GCF_000954235.1 uncultured phage WW-nAnB

GCF_000146025.2 uncultured Termite group 1 bacterium phylotype Rs-D17

The final MALT reference database contained 33,223 genomes and was created using default parameters in *malt-build* (v. 0.4.1). The sequencing data for the ancient pitch sample, two soil control samples and associated extraction and library blanks were de-enriched for human reads by mapping to the human genome (hg19) using BWA aln and excluding all mapping reads. Duplicates were removed with seqkit v.0.7.1^71^ using the ‘rmdup’ function with the ‘–by-seq’ flag. The remaining reads were processed with *malt-run* (v. 0.4.1) where BlastN mode and SemiGlobal alignment were used. The minimum percent identity (–minPercentIdentity) was set to 95, the minimum support (–minSupport) parameter was set to 10 and the top percent value (–topPercent) was set as 1. Remaining parameters were set to default. MEGAN6^15^ was used to visualise the output ‘.rma6’ files and to extract the reads assigned to taxonomic nodes of interest for our sample. A taxon table of the raw MALT output for all samples and blanks, as well as species level read assignments to bacteria, archaea and DNA viruses for the ancient pitch sample are shown in Supplementary Data 5, where reads listed are the sum of all reads assigned to the species node, including reads assigned to specific strains within the species. Reads assigned to RNA viruses were not considered for further analyses, since our dataset consisted of DNA sequences only. Due to the limited number of reads assigned to archaeal species (Supplementary Data 5), we did not consider Archaea in downstream analyses of species identification. To validate the microbial taxa, we aligned the assigned reads to their respective reference genomes and examined the edit distances, coverage distributions, and post-mortem DNA damage patterns (see Supplementary Note 5).

### Pneumococcus analysis

We reconstructed a *S. pneumoniae* consensus genome (Fig. 4) by mapping all reads assigned to *S. pneumoniae* by MALT^16^ to the *S. pneumoniae* TIGR4 reference genome (NC_003028.3). To investigate the presence of multiple strains we estimated the number of heterozygous sites using samtools^57^ mpileup function, filtering out transitions, indels, and sites with a depth of coverage below 10. Coverage statistics of the individual alignments (MQ ≥ 30) were obtained using Bedtools^72^ and plotted using Circos^73^ in 100 bp windows. Mappability was estimated using GEM2^74^ using a k-mer size of 50 and a read length of 42, which is comparable to the average length of the trimmed and mapped reads in the ancient pitch. Virulence genes were identified by assembling the ancient *S. pneumoniae* MALT extracts into contigs using megahit^75^. The contigs were aligned against known *S. pneumoniae* TIGR4 virulence genes in the Virulence Factor Database^17^ (downloaded 22/11–2018) using BLASTn^76^. Only unique hits with a bitscore >200, >20% coverage, and an identity >80% were considered as shared genes (Supplementary Data 6).

To identify all streptococcus virulence factors in the ancient pitch, we aligned the contigs against the full Virulence Factor Database^17^ (downloaded 22/11–2018) using BLASTn^76^ and the same filtering criteria as described above (Supplementary Data 6). To validate the approach we repeated the analysis with five modern oral microbiome samples (SRS014468; SRS019120; SRS013942; SRS015055; SRS014692) from the Human Microbiome Project (HMP)^14^ using only the forward read (R1) (Supplementary Data 6). We find that the number of virulence genes we recovered directly correlates with sequencing depth (Supplementary Fig. 16).

### Holi

For a robust taxonomic assignment of reads aligning to Metazoa (animals) and Viridiplantae (plants), all non-human reads were parsed through the ‘Holi’ pipeline^18^, which was specifically developed for the taxonomic profiling of ancient metagenomic shotgun reads. Each read was aligned against the NCBI’s full Nucleotide and Refseq databases (downloaded November 25th 2018), including a newly sequenced full genome of European hazelnut (*Corylus avellana*, downloaded April 10th 2019)^77^. The alignments were then parsed through a naive lowest common ancestor algorithm (ngsLCA) based on the NCBI taxonomic tree. Only taxonomically classified reads for taxa comprising ≥1% of all the reads within the two kingdoms and a declining edit distance distribution after edit distance 0 were parsed for taxonomic profiling and further validation. To validate the assignments, we aligned the assigned reads to their respective reference genomes and examined the edit distances, coverage distributions, and post-mortem DNA damage patterns (see Supplementary Note 5; Supplementary Data 7).

### Reporting summary

Further information on research design is available in the Nature Research Reporting Summary linked to this article.

## Supplementary information

Supplementary Information

Description of Additional Supplementary Files

Reporting Summary

Supplementary Dataset 1

Supplementary Dataset 2

Supplementary Dataset 3

Supplementary Dataset 4

Supplementary Dataset 5

Supplementary Dataset 6

Supplementary Dataset 7

## Data Availability

The sequencing reads are available for download from the European Nucleotide Archive under accession number PRJEB30280. All other data are included in the paper or available upon request.
