# Supplementary material for: A 5700 year-old human genome and oral microbiome from chewed birch pitch
Source: Nat Commun. 2019 Dec 17;10:5520. doi: 10.1038/s41467-019-13549-9 (PMC6917805; doi:10.1038/s41467-019-13549-9)
Supplement: Supplementary file 1 — Supplementary Information [file 41467_2019_13549_MOESM1_ESM.pdf]

Supplementary Information for

A 5,700 year-old human genome and oral microbiome from chewed birch pitch

Theis Z. T. Jensen[1,2+], Jonas Niemann[1,2+], Katrine Højholt Iversen[3,4+], Anna K. Fotakis[1], Shyam Gopalakrishnan[1], Åshild J. Vågene[1], Mikkel Winther Pedersen[1], Mikkel-Holger S. Sinding[1], Martin R. Ellegaard[1], Morten E. Allentoft[1], Liam T. Lanigan[1], Alberto J. Taurozzi[1], Sofie Holtsmark Nielsen[1], Michael W. Dee[5], Martin N. Mortensen[6], Mads C. Christensen[6], Søren A. Sørensen[7], Matthew J. Collins[1,8], M. Thomas P. Gilbert[1], Martin Sikora[1], Simon Rasmussen[4], Hannes Schroeder[1*]

[1]The Globe Institute, Faculty of Health and Medical Sciences, University of Copenhagen, Copenhagen 1353, Denmark
[2]BioArch, Department of Archaeology, University of York, York YO10 5DD, UK
[3]Department of Bio and Health Informatics, Technical University of Denmark, Kongens Lyngby 2800, Denmark
[4]Novo Nordisk Foundation Center for Protein Research, Faculty of Health and Medical Sciences, University of Copenhagen, Copenhagen 2200, Denmark
[5]Centre for Isotope Research, University of Groningen, Groningen 9747 AG, The Netherlands
[6]The National Museum of Denmark, I.C. Modewegs Vej, Brede, Kongens Lyngby 2800, Denmark
[7]Museum Lolland-Falster, Frisegade 40, Nykøbing Falster 4800, Denmark
[8]McDonald Institute for Archaeological Research, University of Cambridge, Cambridge CB2 3ER, UK

[+]These authors contributed equally to this work.
[*]Correspondence and requests for materials should be addressed to: H.S. (email: hschroeder@bio.ku.dk)

Supplementary Note 1. Site description 3

Supplementary Note 2. Radiocarbon dating 4

Supplementary Note 3. FTIR and GC-MS analysis 5

Supplementary Note 4. Decay rate estimate 6

Supplementary Note 5. Analysis of metagenomic reads 7

Supplementary Note 1. Site description

Theis Z. T. Jensen and Søren A. Sørensen

Syltholm is located in the southern part of Lolland near Rødbyhavn in Denmark (Supplementary Fig. 1). The site covers ca. 187 hectares of land, which up until 1872 was open water. After a series of floods, a reclamation project was undertaken to dam up the area, thus preserving the inundated landscape below[1]. In 2012, Museum Lolland-Falster initiated large scale geological surveys and subsequent archaeological excavations in the area due to the upcoming establishment of the Fehmarn Belt fixed-link tunnel connecting Denmark to Germany. Several sites were selected for full-scale excavations, based on coring, landscape topography as well as investigative excavations. Excavation of the former fjord was significantly constrained by high groundwater levels, which preclude the initial use of mechanical excavators. This was solved by localised drainage for several months[2,3]. To date a total of 21 excavations have been completed. They vary in terms of age, finds intensity as well as preservation of organic material. The vast majority of the site spans from the Late Mesolithic Ertebølle to the Middle Neolithic Funnel Beaker periods. During the time of occupation the area would have been a shallow brackish lagoon protected from the open sea to the south by shifting sandy barrier islands. Human activity in this coastal environment in prehistory is reflected primarily by the finding of large numbers of organic and inorganic artefacts and thousands of faunal remains, many of which include cut-marks[3,4].

In the area of the site where the chewed birch pitch (Supplementary Fig. 2) was found (MLF906-I-II), the stratigraphy consists of 1) a top layer is a heterogeneous matrix of sand separated by thin sections of gyttja, ca. 1-1,5 m in thickness, which indicates several storm events, 2) a thin (5-10 cm) transgression horizon of coarse drift gyttja containing large amounts of molluscs as well as *ex situ* water rolled artefacts of flint and organic material, indicating an erosive milieu, 3) a layer (10-80 cm) of coarse brown gyttja where large amounts of *in situ* organic and inorganic archaeological artefacts and other material was uncovered, indicating a sheltered environment, and 4) a bottom layer of glacial till, which consists of blue clay. The glacial topography consists of several small depressions, which in the Ertebølle and Neolithic periods would, at certain places, gradually have been filled with organic matter forming gyttja.

Several hundred unpublished AMS dates from MLF906-II, including the ones presented in this manuscript (Supplementary Fig. 3) indicate that the area was frequented by people more or less continuously from the Late Mesolithic until Middle Neolithic. Continued artefact depositions seem to have been carried out at the site, as dates obtained from organic material, such as bone, antler or wood, found in small confined areas span nearly 1,000 years. During the earlier (Mesolithic) phase, the deposits are dominated by wild taxa, including red deer (*Cervus elaphus*), roe deer (*Capreolus capreolus*), and pig (*Sus sp.*), as well as ox (*Vulpes vulpes*), otter (*Lutra lutra*), and wildcat (*Felis silvestris*), although large numbers of domestic dog (*Canis familiaris*) remains have also been found[5]. From around 5,800 BP other domesticated species (e.g. *Bos taurus*) also start to appear, but keep being dominated by wild taxa (see Supplementary Fig. 3).

Supplementary Note 2. Radiocarbon dating

Michael W. Dee

Radiocarbon dating was performed on ca. 10 mg of the birch pitch, following an acid-base-acid pretreatment[6]. First, the sample was treated with 4% HCl (80°C) and then rinsed to neutrality with ultra-pure water. Second, a basic solution 1% NaOH (RT) was applied, and the reaction vessel rinsed again to neutrality. Finally, a further acid step was applied using 4% HCl (80°C) to ensure no atmospheric CO_2 absorbed during the alkaline phase remained in the reaction vessel. After a last rinse to neutrality, the product was thoroughly air dried. An aliquot of ca. 4 mg was then weighed into a tin capsule for combustion in an Elementar IsotopeCube NCS Elemental Analyser (EA). The EA was coupled to an Isoprime 100 Isotope Ratio Mass Spectrometer, which allowed the $\delta^{13}C$ value of the sample to be measured, as well as a fully automated cryogenic system that trapped the liberated CO_2 into an airtight vessel. The vessel was manually transferred to a vacuum manifold, where a stoichiometric excess of H_2(g) (1: 2.5) was added, and the sample CO_2(g) reduced to graphite over an Fe(s) catalyst. The graphite was pressed into a cathode for radioisotope analysis in an MICADAS IonPlus accelerator mass spectrometer. The MICADAS generated an estimate of the ^{14}C:^{12}C ratio that was close to ±1‰, and from this data, and in accordance with all standard operations and conventions, the ^{14}C date (in yrs BP) was calculated. Thc calendar date range (years cal BP) was computed using the calibration program OxCal (v 4.3)[7].

Inaccuracy in ^{14}C dating largely arises from processes that occur before the sample reaches the laboratory. Misassociation of sample and context, or factors which can make substances ‘appear older’ such as marine/dietary reservoir effects or material reuse, are the most common. Enriching effects, which would cause the age to be too young, are negligible in the natural world. However, human error in the ^{14}C laboratory can introduce both younger or older contamination. To guard against such sources of inaccuracy, the radiocarbon laboratory in Groningen regularly analyzes reference samples, including full pretreatments and measurements on materials of independently known age.

Supplementary Note 3. FTIR and GC-MS analysis

Martin N. Mortensen and Mads C. Christensen

For the FTIR analysis ca. 1 mg of sample was ground with KBr (Fischer Scientific, IR Grade), pressed into a pellet and measured on a Perkin Elmer Spectrum 1000 FT-IR spectrometer. The FTIR spectra for the Syltholm pitch and a modern birch sample are shown in Supplementary Fig. 4. For the GC-MS analysis, ca. 0.5 mg sample was hydrolysed in methanolic KOH (Merck) and extracted with GC-grade tert-Butyl methyl ether (MTBE) after acidification. The extract was methylated using diazomethane (Sigma-Aldrich)[8]. 1 µl of this solution was injected on a Bruker SCION 456 GC-TQMS system equipped with a Programmable Temperature Vaporizer that was held at 64°C for 0.5 min, raised to 315°C at 200°C min^{-1} and held at that temperature for 40 min. The split ratio was high during the first 0.5 min and then switched to 5. The GC column was a Restek Rtx-5 capillary column (30 m, 0.25 mm ID, 0.25 µm) and the He flow rate was 1 cm^3 min^{-1}. The GC oven temperature was held at 64°C for 0.5 min, then raised to 190°C at 10°C min^{-1} and then onto 315°C at 4°C min^{-1} and held at that temperature for 15 min. The EI (electron ionisation) ion source was held at 250°C and the ionisation potential was -70 eV. The mass spectrometer was operated in the full scan mode from *m*/*z* 45 to *m*/*z* 800. The GC-MS chromatograms for the Syltholm pitch and the betulin and lupeol references are shown in Supplementary Fig. 5.

Supplementary Note 4. Decay rate estimate

Morten E. Allentoft and Hannes Schroeder

To investigate the rate of human DNA degradation in the ancient pitch sample we examined the DNA read length distributions of the mapped reads, using a previously published method[9]. The distribution follows a typical pattern of degraded DNA with an initial increase in the number of reads towards longer DNA fragments, followed by a decline. We observe that the declining part of the distribution follows an exponential decay curve (R^2=0.99), as expected if the DNA had been randomly fragmented over time. Deagle et al.[10] showed that the decay constant (λ) in the exponential equation represents the fraction of broken bonds in the DNA strand (the damage fraction) and that 1/λ is the average theoretical fragment length in the DNA library. By solving the equation, we obtain a DNA damage fraction (λ) of 3.4%, which corresponds to a theoretical average fragment length (1/λ) of 29 bp (Table S2). We note that this is not directly comparable to the observed average length, which is affected by lab methods and sequencing technology. If the DNA is found in a stable matrix long term DNA fragmentation can be expressed as a rate and the damage fraction (λ, per site) can be converted to a decay rate (*k*, per site per year), when the age of the sample is known. By applying an estimated age of 5,700 years for the Syltholm pitch, the corresponding DNA decay rate (*k*) is $5{,}96^{-06}$ breaks per bond per year, which corresponds to a molecular half-life of 1,162 years for a 100 bp DNA fragment. This means that after 1,162 years (post cell death), each 100 bp DNA stretch will have experienced one break on average. This estimated rate of DNA decay for the pitch sample seems within the expected age for DNA preserved in a stable matrix in a temperate climate zone. For example, the rate is close to that observed in the La Braña sample[11], preserved at similar temperatures as the pitch sample (Supplementary Table 2). By contrast, the DNA decay in human remains from warmer climates is much faster[12]. Although these calculations are only based on a single sample, the results suggest that ancient mastics provide remarkable conditions for molecular preservation.

Supplementary Note 5. Analysis of metagenomic reads

Jonas Niemann and Hannes Schroeder

Accurate taxonomic classification of complex metagenomic datasets can be challenging, especially if closely related species are present in the sample or as environmental contaminants[13]. Additionally, robust classification can be complicated if reference databases are incomplete or sequencing effort is insufficient. A further complication is that, in some cases, reference databases contain poor quality genomes with contaminant sequences, which can lead to incorrect assignments[14,15]. While specific pipelines for the taxonomic classification of ancient metagenomic datasets have been developed[16,17], further validation is often necessary to exclude the possibility of false positive (misidentified) assignments. Methods used for validation include confirming the presence of ancient DNA damage patterns, evaluating edit distances, and assessing coverage distributions[18,19].

To test the robusticity of our pipelines[16,17], we performed two in silico experiments using archaeological and environmental samples as controls. First, we ran MetaPhlan2[20] and MALT[17] on two soil samples from the site and show that they have a completely different microbial composition from the ancient pitch (Fig. 4; Supplementary Data 4; Supplementary Data 5). We then ran Holi[16] on the same controls and, using the same criteria as for the ancient sample, did not retrieve any reads that could be assigned to the eukaryotic taxa we identified in the ancient pitch (Supplementary Data 7). Second, we ran the Holi pipeline[16] on a previously published dataset[12] generated from an ancient tooth (~33 million reads with an average length of 69 bp) to test whether some of our results might be false positives resulting from reference genomes being contaminated with DNA from other species, especially human DNA. Using the same criteria as the ones we applied in the present study, we did not identify any of the taxa we identified in the ancient pitch.

Independent validation of taxonomic assignments

To validate the taxonomic assignments of the metagenomic reads recovered from the ancient pitch, we aligned the assigned reads to their respective reference genomes and examined the edit distances, coverage distributions, and post-mortem DNA damage patterns[18,19]. For the bacterial taxa identified by MALT, we chose to further investigate bacterial species with ≥10,000 assigned reads (including strain specific reads). We then aligned the taxon-specific MALT extracts to their respective reference genomes that we obtained from the NCBI assembly database (Supplementary Data 5). The sequences were aligned using *bwa aln*[21] and PCR duplicates were removed using Picard Tools v.2.13.2[22]. MapDamage v.2.0.9[23] was used to estimate deamination rates (Supplementary Fig. 8). The breadth and depth of coverage were calculated with *bedtools* v.2.27.1[24] and visualised with *Circos* v.0.69-6[25] using a window size of 100 bp (Supplementary Fig. 9). Edit distances for all reads and filtered for PMD score ≥1 were extracted from the bam files with samtools view[21] and PMDtools[26] and plotted in R v.3.4.1[27] (Supplementary Fig. 10). The negative difference proportion (-Δ%) was calculated using only reads with PMD score ≥1. This metric was first introduced by Hübler et al.[19] and is a measure of the decline in the edit distance distribution, with a -Δ% value of 1

indicating a strictly declining distribution. Correct taxonomic assignments generally result in a continuously declining edit distance distribution, which reflects the fact that most of the aligned reads show no or only few mismatches, mostly resulting from aDNA damage or divergence of the ancient genome from the modern reference. By contrast, mapping to an incorrect reference tends to result in an increased number of mismatches, which is reflected in the edit distance distribution[19]. For the microbial taxa, we report species-specific assignments with a -Δ% value >0.8 to account for the possibility of cross-alignments due to horizontal gene transfer and the presence of closely related microbial species in the sample.

The Human Oral Microbiome Database (HOMD) was referred to in order to classify bacterial species as belonging to the human oral/respiratory microbiome or as environmental. Of the 64 most abundant bacterial species identified in the ancient pitch (Supplementary Data 5), four are known contaminants originating from lab reagents (*Delftia* spp.), which are also evident in the extraction blanks (Supplementary Data 5), while seven (*Pseudomonas stutzeri*, *Hydrogenophaga sp.* RAC07, *Leptospira alstonii*, *Ramlibacter tataouinensis*, *Thalassolituus oleivorans*, *Achromobacter spanius*, *Pseudomonas aeruginosa*) are likely derived from the environment. None of these 11 species showed the characteristic damage patterns of ancient DNA and were, therefore, not included in further analyses. The remaining 53 bacterial species are predominantly found in the oral cavity and the upper respiratory tract (see Table 1; Supplementary Data 5).

Among the viral species identified we chose to further authenticate reads assigned to the Epstein-Barr virus (*Human gammaherpesvirus 4*) (Supplementary Fig. 11), since it is the only non-bacteriophage viral taxon to which ≥200 reads were assigned. Viruses have considerably smaller genomes than bacteria and were therefore subject to a lower threshold of assigned reads.

The plant and animal taxa identified by Holi[16] were validated by evaluating sequence identity through edit distance distributions, evenness of coverage, and the presence of post-mortem DNA damage patterns as described above after extracting family level reads for each taxon and aligning them to their respective reference genome. For taxa with low coverage, we used bedtools[24] to calculate the proportion of mapped bases with a coverage >1× as an alternative way of assessing evenness of coverage (Supplementary Data 7). Using these criteria, we identified four taxa (*Anas platyrhynchos, Anser cygnoides, Betula pendula, Corylus avellana*) which showed characteristic ancient DNA damage patterns and a strictly declining edit distance distribution (Supplementary Fig. 12-14 and Supplementary Data 6). However, further analysis using mitochondrial (mtDNA) genomes as reference yielded only 291 reads aligning to the *A. cygnoides* mtDNA in contrast to 2,541 for the *A. platyrhynchos* (mallard) mtDNA, with >99% of bases covered and nearly 10× average depth of coverage (Supplementary Data 6). Furthermore, the edit distance distribution for the *A. cygnoides* mtDNA (Supplementary Fig. 13) is not declining, suggesting a poor match. We therefore excluded *A. cygnoides* as a likely false positive assignment.

As a further validation step and to assess whether reads from multiple taxa might have been misassigned to a single species, we examined the number of multiallelic sites in the 10× haploid mallard mtDNA. In haploid genomes (i.e. bacterial genomes or mtDNA), the vast

majority of variable sites should be monoallelic, so that a large number of multiallelic sites might be indicative of multiple species or strains being present[13]. To assess the allele frequency distribution for the 10× mallard mtDNA, we rescaled the base qualities of the mallard mtDNA reads according to their likelihood of being damaged using mapDamage v.2.0.9[23] and called variants using samtools[21] mpileup function using a minimum depth of 10. The allele frequency distribution follows a normal distribution with a mean of ~0.5 indicating the presence of two haplotypes (Supplementary Fig. 15). This was confirmed by visual inspection of the alignment in IGV v.2.3.9[28]. However, rather than indicating the presence of two different taxa, we believe that this might indicate the presence of two individuals and it is not inconceivable that two or more individuals were consumed. This is supported by the fact that the only other Anatidae species with a significant number of reads identified by Holi[16] was the swan goose (*A. cygnoides*). However, as discussed above, we excluded this taxon based on the poor level of sequence identity with the *A. cygnoides* mtDNA as evident in the edit distances (Supplementary Fig. 13). We were unable to evaluate haploidy for the two plant taxa (*Betula pendula* and *Corylus avellana*) since the depth of coverage of the chloroplast DNA was too low.

We also identified 3,213 reads that could be assigned to the human endoparasite *Spirometra erinaceieuropaei* (tapeworm). However, although the reads appear to be ancient, coverage was not even (>60% of mapped bases >1× despite an average depth of coverage of only 0.000025×) suggesting that they are likely false positive alignments perhaps due to the presence of contaminant (human) sequences in the reference (Supplementary Data 7). Recent studies[15] have shown that public genome assemblies of parasitic worms can be contaminated with DNA from the host species, other species that are commensal in the host, or laboratory contaminants, highlighting the need for curating public reference genome databases[29].

Lastly, we acknowledge that it is possible that some of the eukaryotic taxa we report (e.g. mallard) may have come from the environment as opposed to the diet. However, since the vast majority of the DNA we retrieved from the ancient pitch appears to be endogenous (i.e. either human or from the oral cavity), we find this to be unlikely and we believe that it is more likely that the taxa we report derived from the diet.

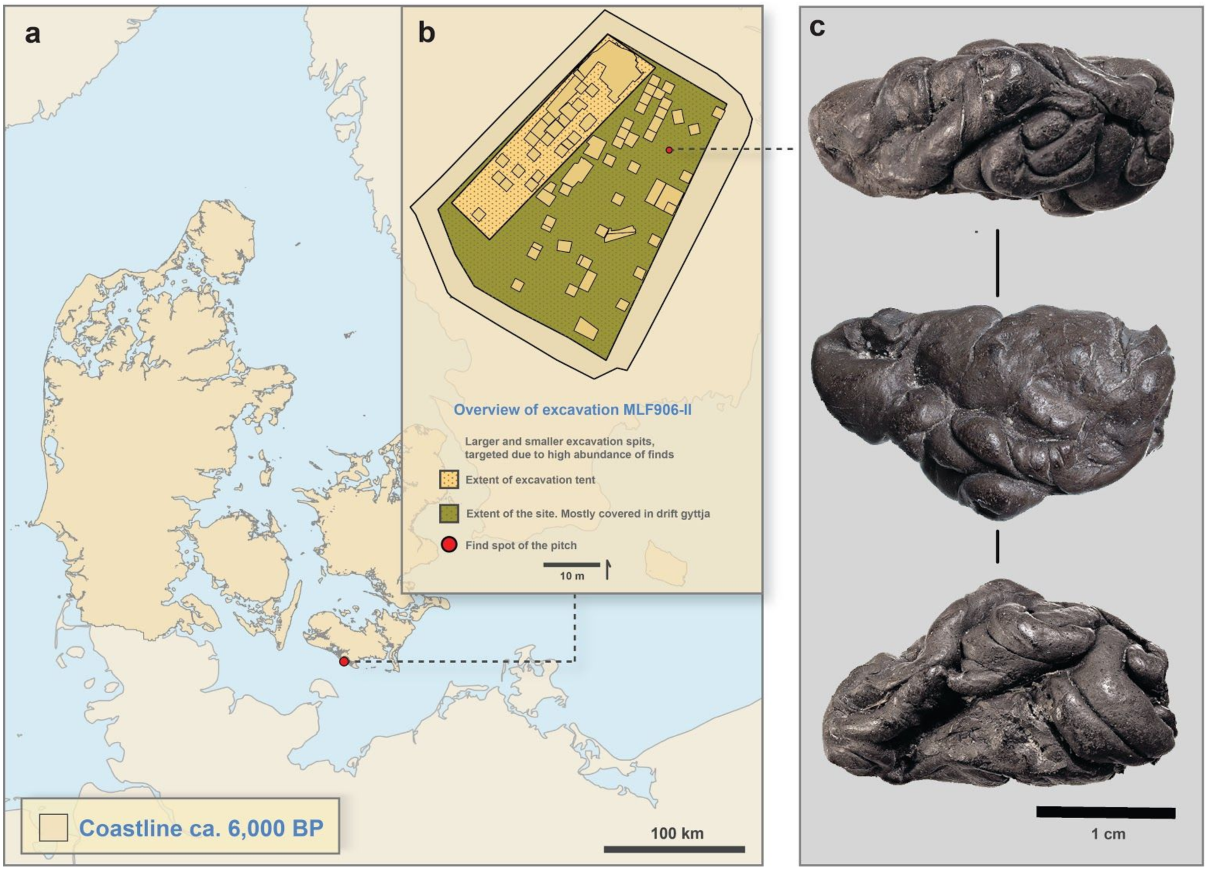

Supplementary Figure 1. a, Map of Denmark showing the location of Syltholm on the island of Lolland (map created using data from Astrup[30]). **b,** GIS site plan of the excavation and findspot of the birch pitch. **c,** photograph of the birch pitch.

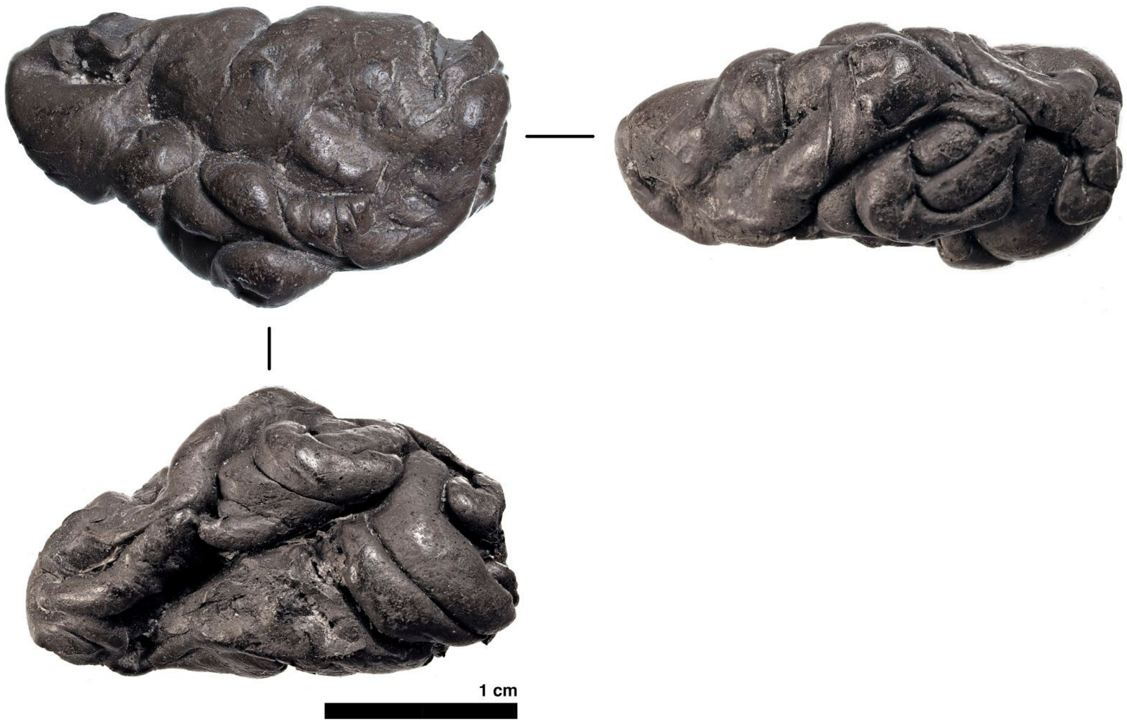

Supplementary Figure 2. Close-up photograph of the Syltholm pitch.

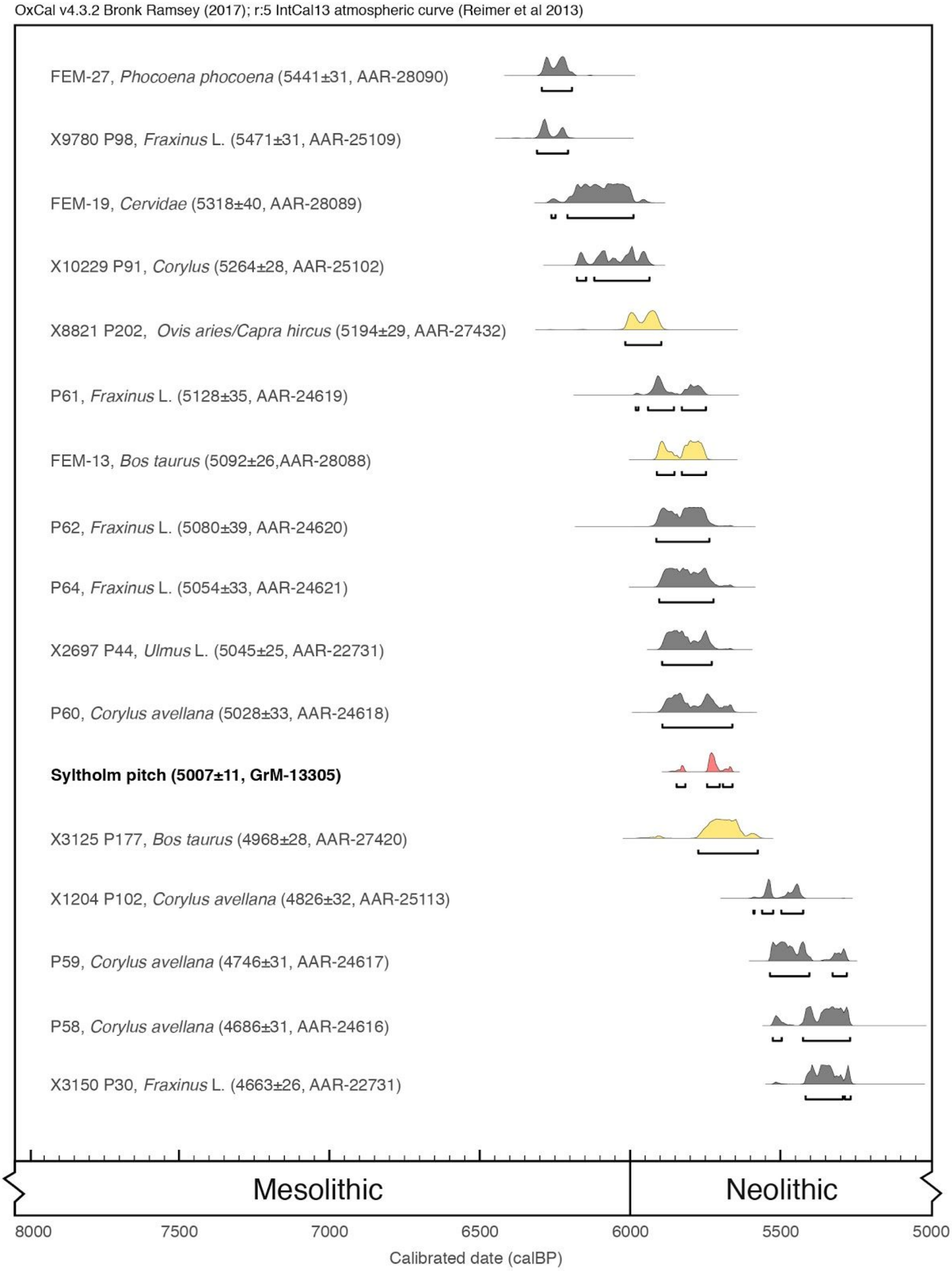

Supplementary Figure 3. Radiocarbon chronology for Syltholm site MLF906-II based on a series of 17 calibrated radiocarbon dates, including the birch pitch (marked in red). Samples from domesticated species are marked in yellow.

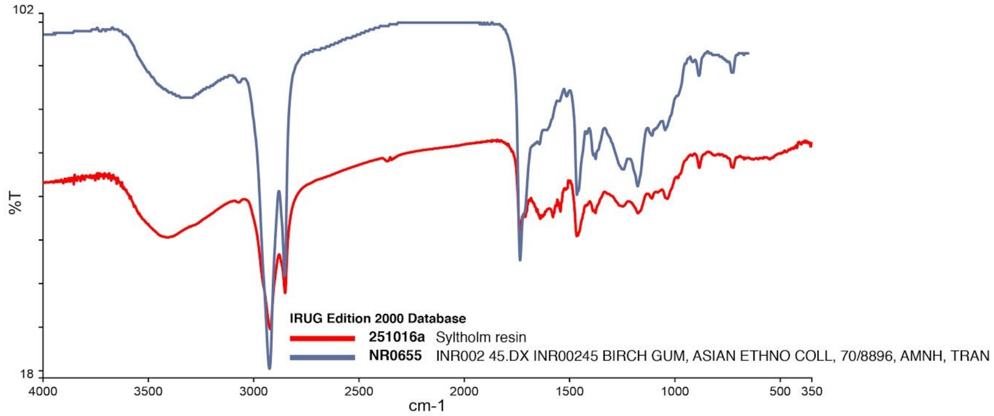

Supplementary Figure 4. FT-IR spectra of the Syltholm pitch and a modern birch sample.

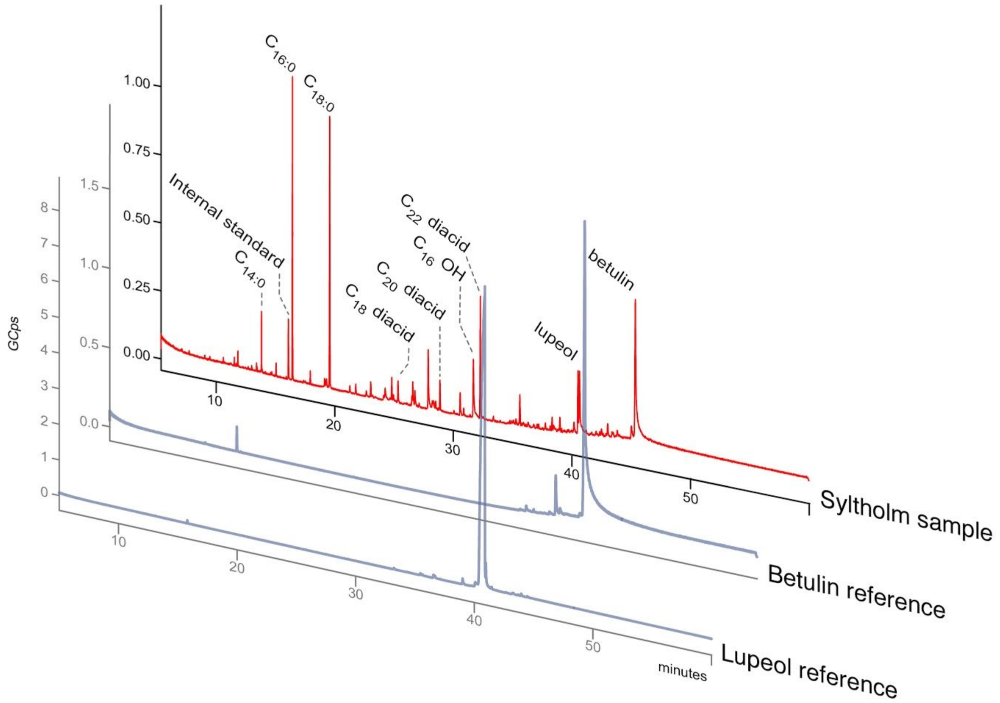

Supplementary Figure 5. GC-MS chromatograms of the Syltholm sample (back), betulin reference (middle) and lupeol reference (front).

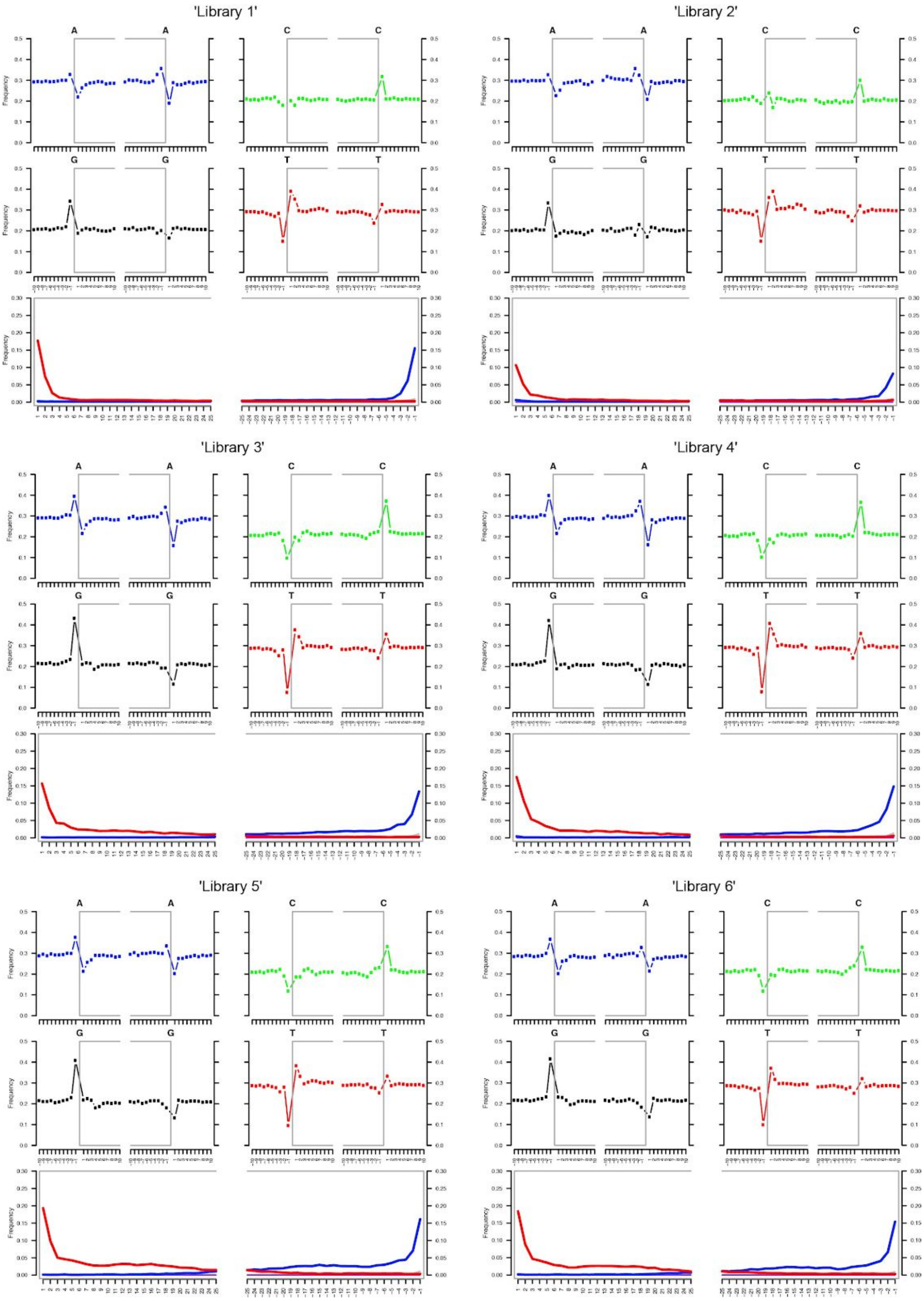

Supplementary Figure 6. MapDamage[23] plots for reads mapping to the human reference genome (hg19), by library.

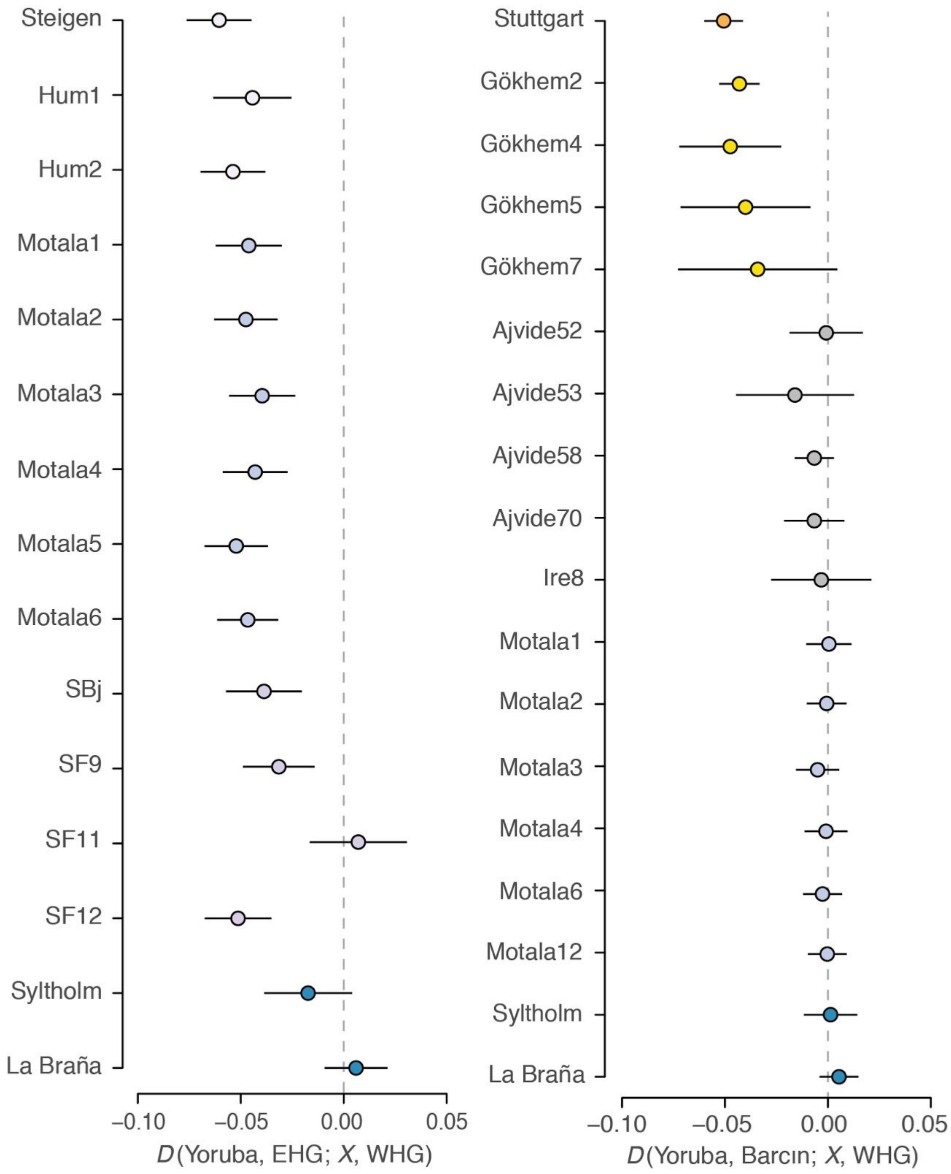

Supplementary Figure 7. *D*-statistics of the form *D*(Yoruba, EHG/Barcın; *X*, WHG) testing whether "*X*" forms a clade with WHG to the exclusion of EHG and Neolithic farmers (represented by Barcın), respectively. Error bars show three block-jackknife standard errors. Data are shown in Supplementary Tables 7 and 8.

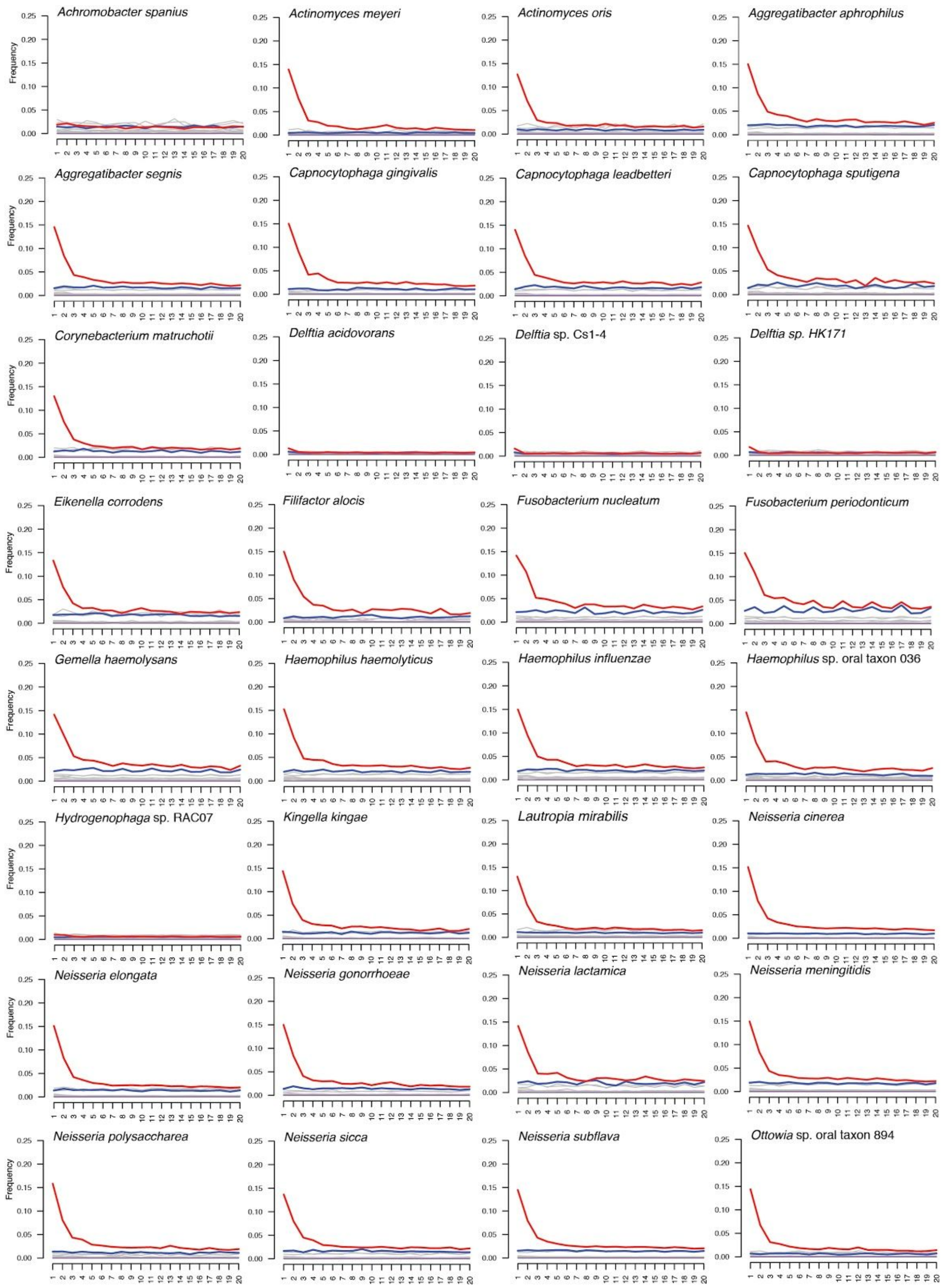

Supplementary Figure 8. MapDamage[23] plots for bacterial taxa with >10,000 assigned reads recovered from the Syltholm pitch.

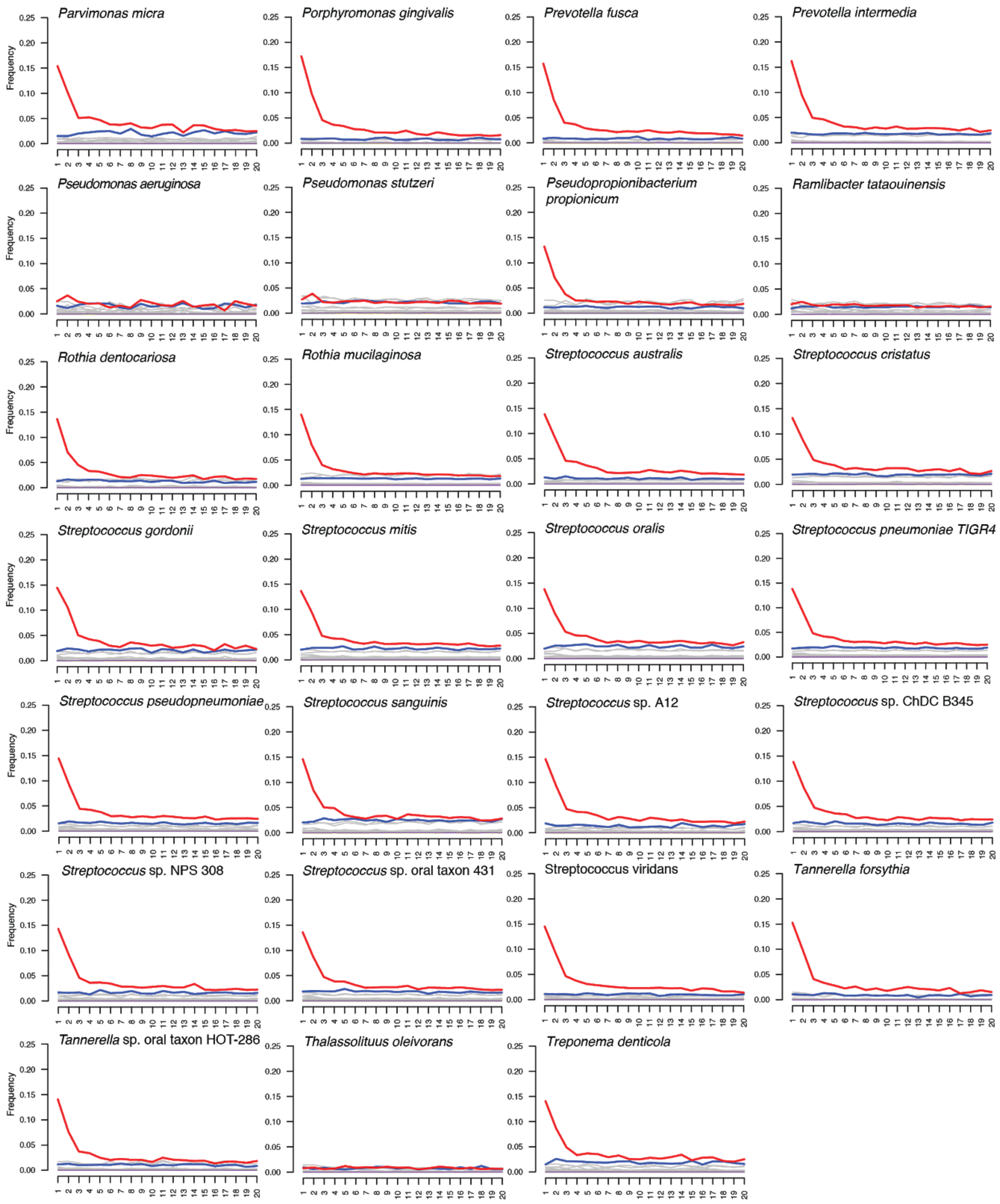

Supplementary Figure 8 ctd. MapDamage[23] plots for bacterial taxa with >10,000 assigned reads recovered from the Syltholm pitch.

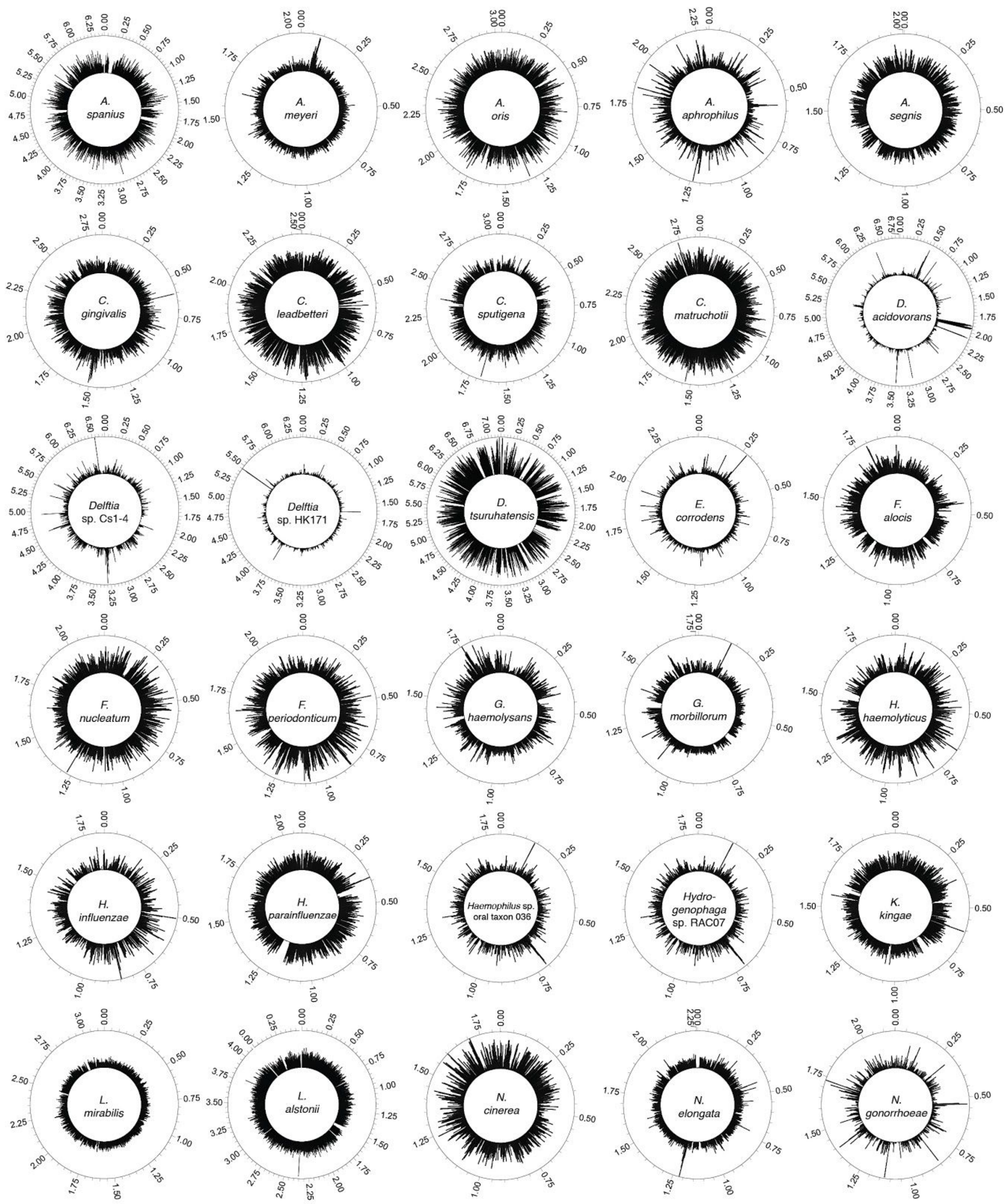

Supplementary Figure 9. Coverage plots for bacterial taxa recovered from the Syltholm pitch.

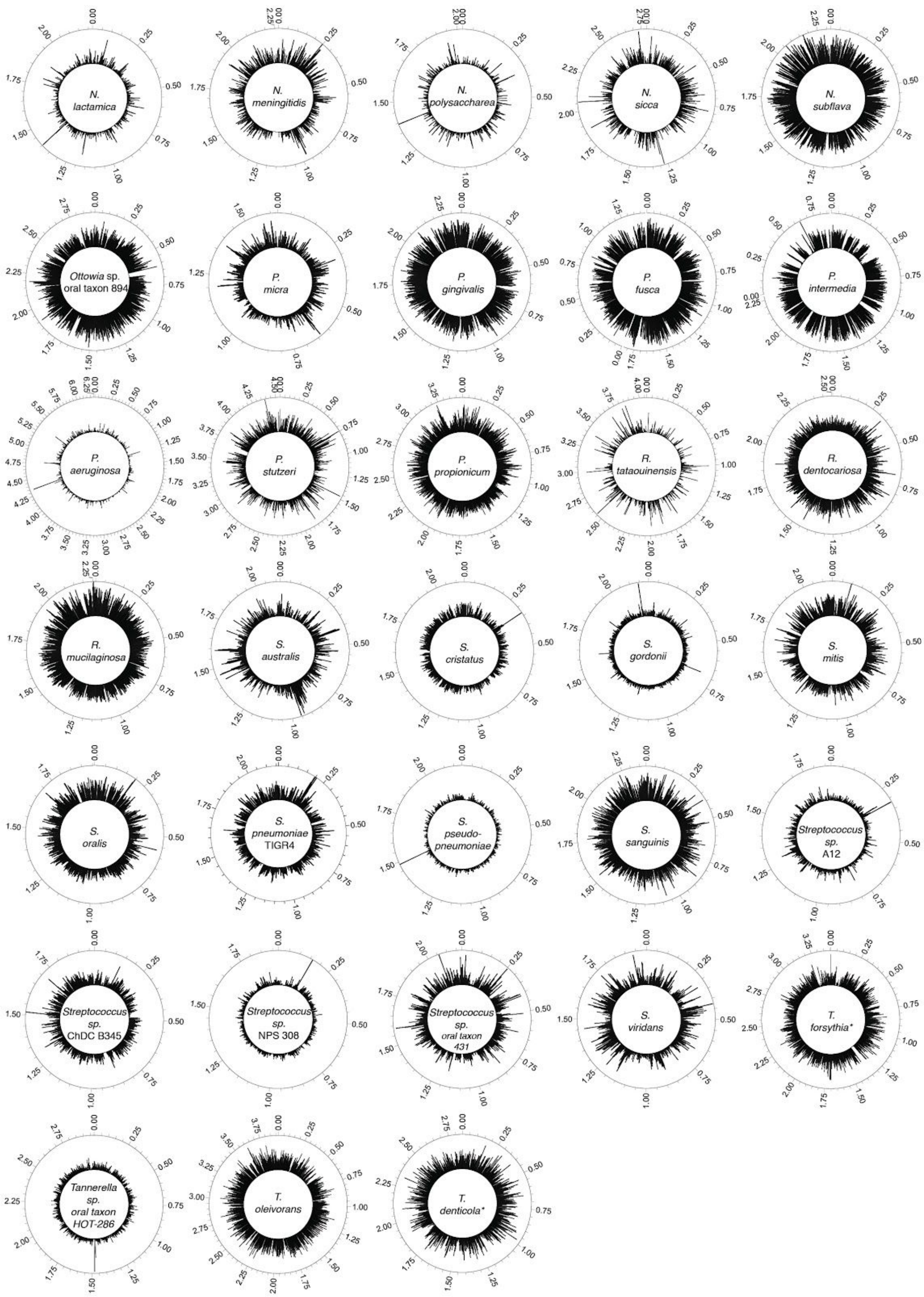

Supplementary Figure 9 ctd. Coverage plots for bacterial taxa recovered from the Syltholm pitch.

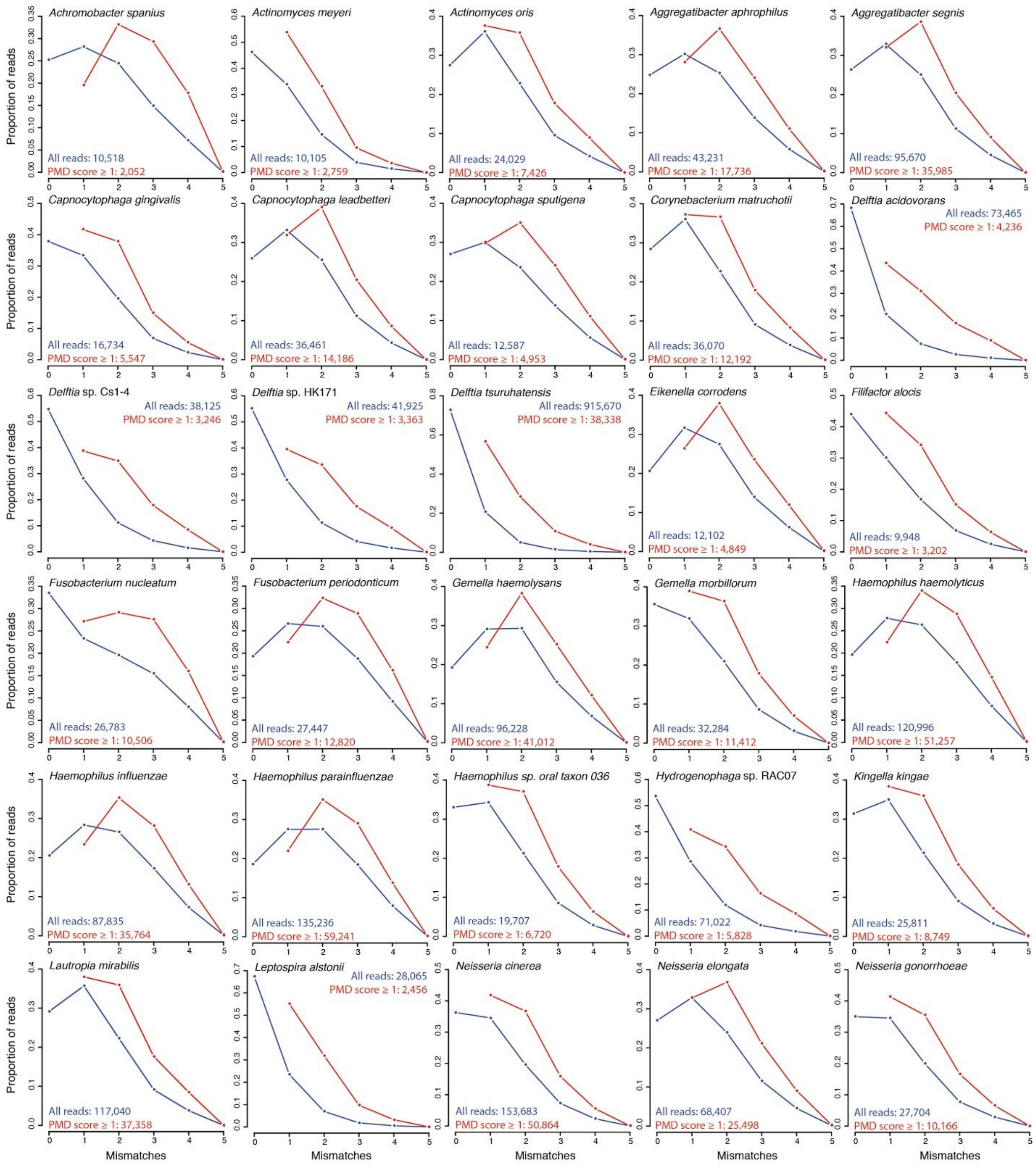

Supplementary Figure 10. Edit distance distributions of all reads (blue) and reads filtered for post-mortem damage (PMD≥1) (red) for bacterial taxa with >10,000 assigned reads recovered from the Syltholm pitch.

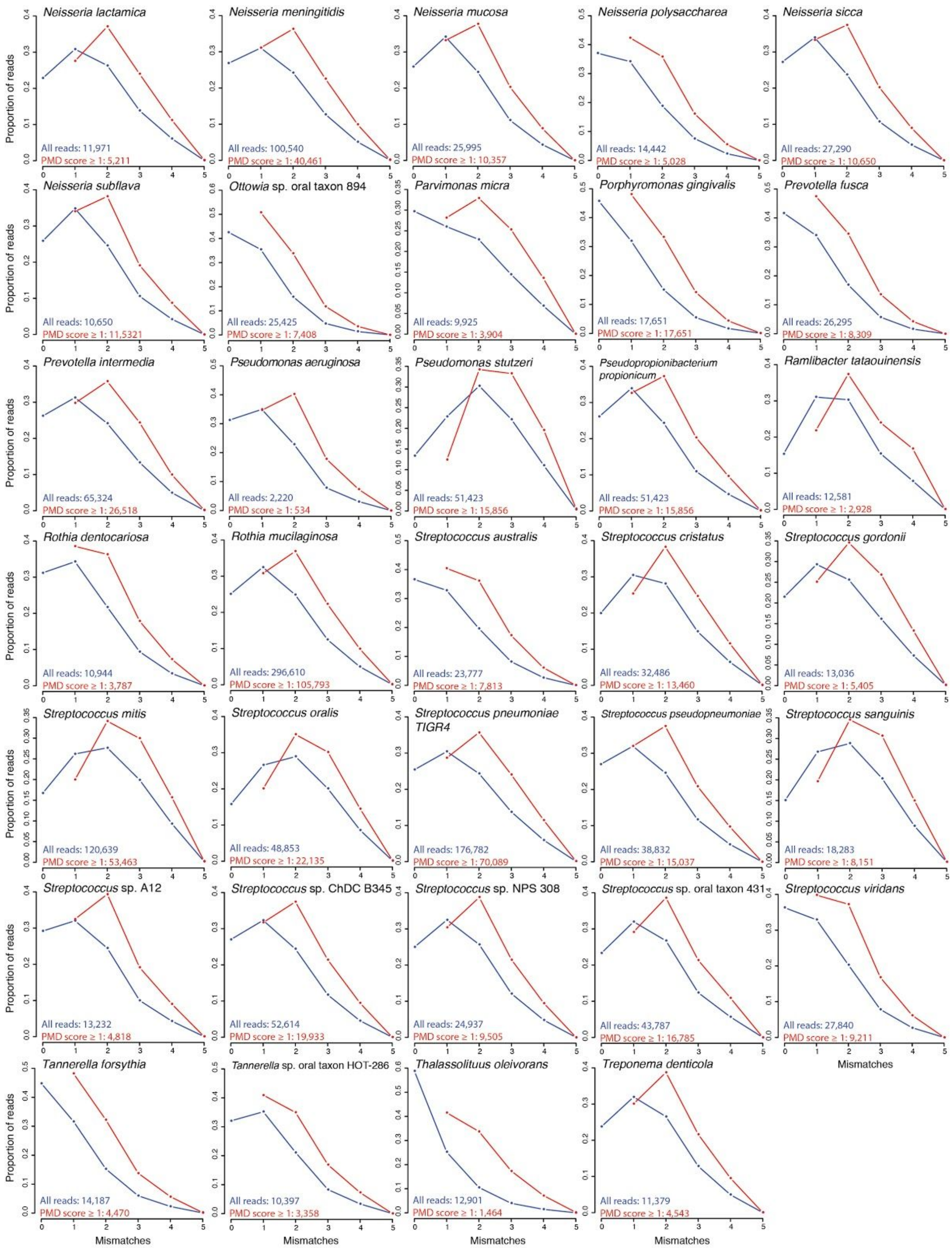

Supplementary Figure 10 ctd. Edit distance distributions of all reads (blue) and reads filtered for post-mortem damage (PMD≥1) (red) for bacterial taxa with >10,000 assigned reads recovered from the Syltholm pitch.

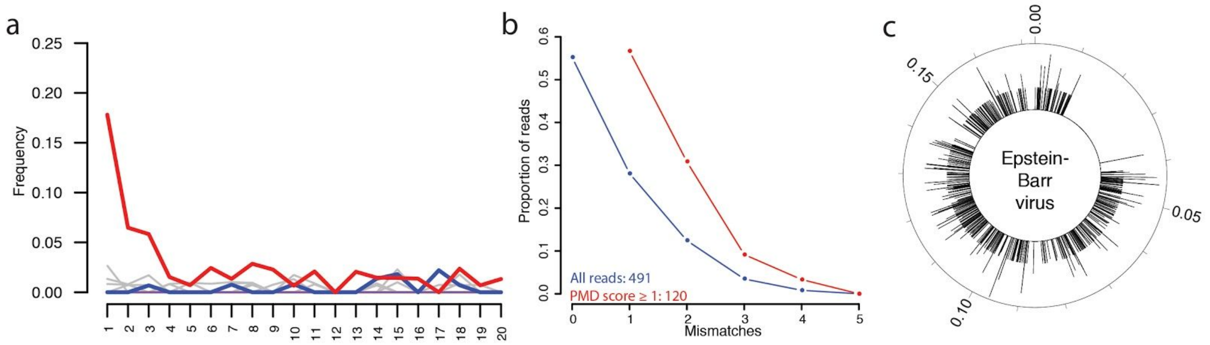

Supplementary Figure 11. MapDamage[23] plot (a), edit distance distribution (b), and coverage plot (c) for reads mapping to Epstein-Barr virus.

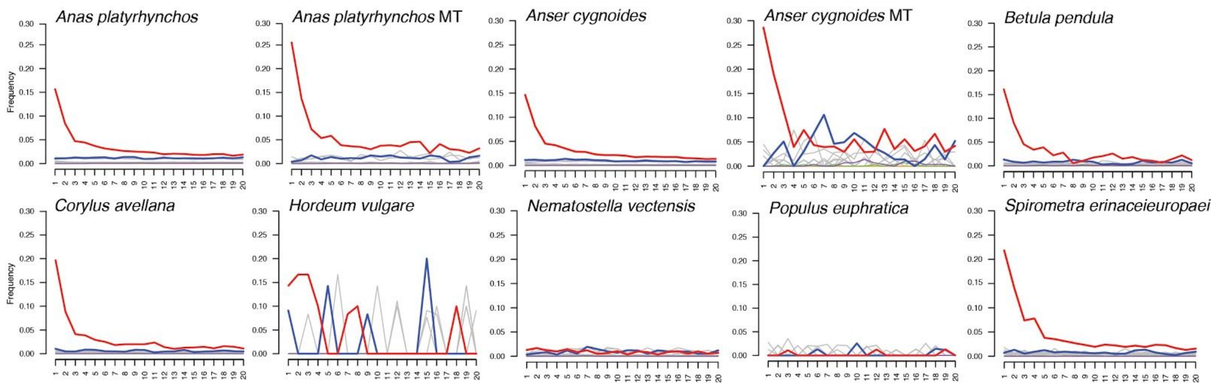

Supplementary Figure 12. MapDamage[23] plots for reads mapping to Metazoa (animals) and Viridiplantae (plants) in the ancient pitch sample. Note the absence of characteristic ancient DNA damage patterns for poplar (*Populus euphratica)*, starlet sea anemone (*Nematostella vectensis)* and barley (*Hordeum vulgare*).

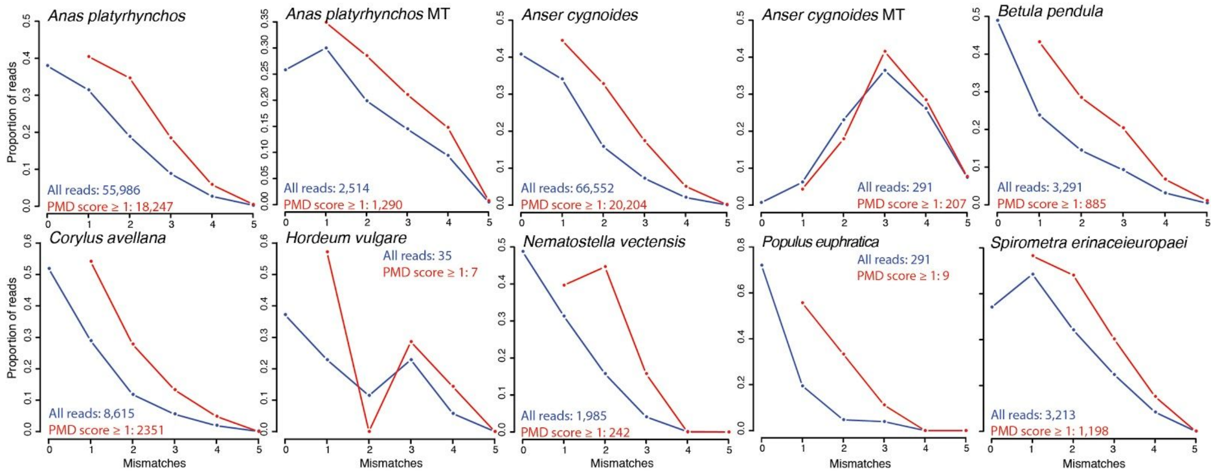

Supplementary Figure 13. Edit distance distributions of all reads from the ancient pitch assigned to Metazoa (animals) and Viridiplantae (plants). Reads filtered for post-mortem damage (PMD≥1) are shown in red.

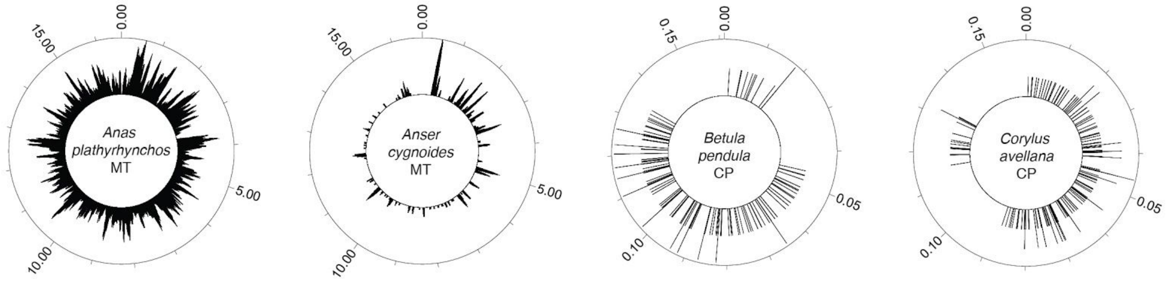

Supplementary Figure 14. Coverage plots for eukaryotic taxa in the ancient pitch sample with more than 100 reads aligning to the chloroplast/mitochondrial genome. For poplar (*Populus euphratica)*, starlet sea anemone (*Nematostella vectensis)* and the tapeworm (*Spirometra erinaceieuropaei*) no fragment aligned to its cpDNA or mtDNA, while for barley (*Hordeum vulgare*) only 15 fragments aligned to its cpDNA. The gaps in the chloroplast DNA (cpDNA) represent inverted repeats, which are very similar to each other, although not completely identical.

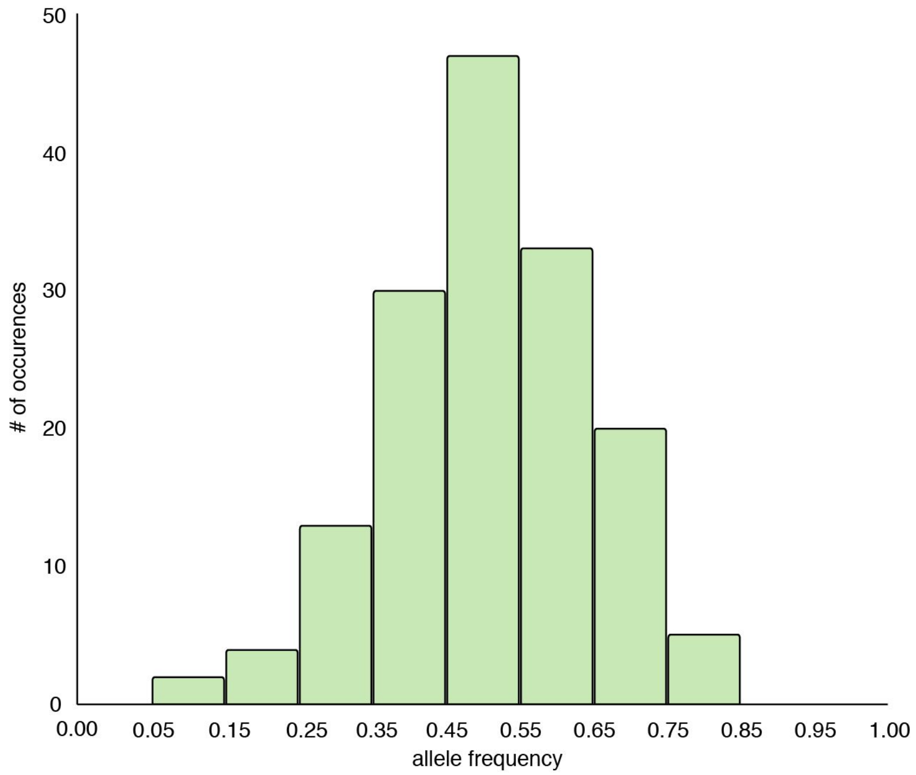

Supplementary Figure 15. Allele frequency distribution of single nucleotide variants in the 10× mallard (*A. platyrhynchos*) mtDNA genome recovered from the ancient pitch. The symmetric distribution suggests the presence of two haplotypes present in equal abundance.

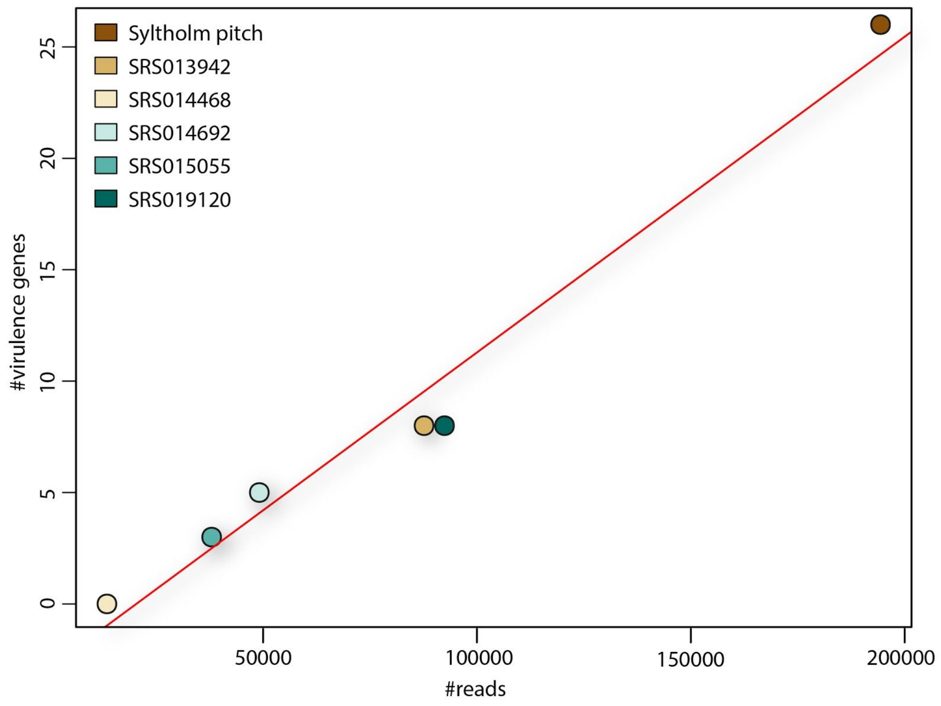

Supplementary Figure 16. The number of virulence genes identified in the ancient pitch sample and five human oral microbiome samples from the HMP[31].

Supplementary Table 1. Screening results for six different DNA extracts from the Syltholm pitch, extraction blank, and two soil control samples from the site.

sample	weight	method	yield (ng)[1]	hg19 reads[2]	% dupl.[3]	% end.[4]	fragment length[5]	C-T 5' (%)[6]
1	54 mg	1	4.7	449,096	9.1	3.7	56.1	17.4
2	52 mg	1	17.3	2,189,982	44.1	24.1	55.4	10.4
3	44 mg	2	6.8	2,754,931	6.3	56.5	59.9	15.0
4	48 mg	2	6.1	3,895,487	9.0	55.1	59.8	17.0
5	32 mg	3	0.3	63,390	57.8	1.6	62.3	19.3
6	24 mg	3	0.3	144,681	46.5	4.3	64.5	18.5
Control 1	~2 g	2	61.4	450	46.4	<0.1	50.8	18.8
Control 2	~2 g	2	58.2	401	50.2	<0.1	46.0	8.3
NTC	N/A	2	N/A	140	72.0	0.5	57.1	13.9

[1]Total DNA yields (ng) measured using the Agilent 4200 TapeStation; [2]Number of reads that could be uniquely mapped to the human reference genome (hg19) after removing duplicates; [3]Fraction of duplicate reads in the sample (in percent); [4]Endogenous human DNA content (in percent); [5]Average fragment length (in bp); [6]Deamination rate at 5' ends of DNA fragments (in percent)

Supplementary Table 2. Deep-sequencing results for the Syltholm pitch.

hg19 reads[1]	end. content[2]	fragment length[3]	C-T 5'[4]	mtDNA contamination[5]	>1X[6]	DoC[7]	mtDNA hg[8]
120,585,267	31.2%	59.9 bp	16.2%	1-3%	78.9%	2.3x	K1e

[1]Number of reads that could be uniquely mapped to the human reference genome (hg19) after removing duplicates and filtering for mapping quality (MAPQ ≥30); [2]Endogenous human DNA content (in percent); [3]Average fragment length (in bp); [4]Deamination rates at 5' ends of DNA fragments (in percent); [5]MtDNA based contamination estimates determined using Schmutzi[34]**;** [6]Genome coverage (in percent); [7]Average depth of genome coverage; [8]Mitochondrial DNA haplogroup.

Supplementary Table 3. Molecular decay rates (*k*, per site per year) for the Syltholm genome and other previously published ancient genomes from different contexts[11,12,32,33].

Sample	Age (yrs BP)	Temp. (°C)	λ	*k*	*k*, 100 bp	half-life (yrs), 100 bp
Taino (The Bahamas)	1,000	20	0.016	1.60^{-05}	1.60^{-03}	434
Syltholm (Denmark)	5,700	8.5	0.034	5.96^{-06}	5.96^{-04}	1,162
La Braña (Spain)	7,500	8.1	0.033	4.40^{-06}	4.40^{-04}	1,576
Kennewick (WA, USA)	9,000	12.5	0.017	1.89^{-06}	1.89^{-04}	3,670
Anzick (MT, USA)	12,785	4.8	0.018	1.41^{-06}	1.41^{-04}	4,916

Supplementary Table 4. *F*-statistics of the form f_4(Yoruba, *X*; EHG, WHG) measuring the amount of shared genetic drift between different ancient genomes (*X*), EHG and WHG.

Pop2 (X)	f_4-stat	SE	Z	BABA	ABBA	SNPs
Syltholm	0.011917	0.000698	17.063	6,281	4,903	115,687
La Braña	0.012022	0.000525	22.894	29,695	23,219	538,716
Hum1	-0.001431	0.000644	-2.224	9,966	10,262	207,167
Hum2	-0.001152	0.000592	-1.947	26,029	26,646	536,119
Steigen	-0.001494	0.000565	-2.645	20,418	21,047	421,170
Motala1	0.00216	0.000575	3.755	17,719	16,950	355,954
Motala2	0.003681	0.000546	6.747	22,397	20,771	441,690
Motala3	0.002856	0.000529	5.396	13,191	12,427	267,396
Motala4	0.003171	0.000578	5.484	22,361	20,955	443,456
Motala6	0.002229	0.000554	4.023	18,922	18,073	380,891
Motala12	0.002848	0.000545	5.223	25,448	24,005	506,761

Supplementary Table 5. *F*-statistics of the form f_4(Yoruba, *X*; NEO, WHG) measuring the amount of shared genetic drift between different ancient genomes (*X*), WHG, and Neolithic farmers (represented by Barcın).

**Pop2 (*X*)**	**f_4-stat**	**SE**	**Z**	**BABA**	**ABBA**	**SNPs**
Syltholm	0.019419	0.000586	33.11	7,292	4,941	121,065
La Braña	0.017952	0.000436	41.145	34,006	23,859	565,167
Motala1	0.012127	0.000476	25.488	20,388	16,010	361,017
Motala2	0.013533	0.000425	31.864	25,789	19,700	449,904
Motala3	0.011315	0.00042	26.961	14,803	11,765	268,527
Motala4	0.012719	0.000444	28.662	25,548	19,828	449,704
Motala6	0.012387	0.000425	29.156	21,691	16,930	384,398
Motala12	0.012751	0.000426	29.936	29,548	22,926	519,374
Ajvide52	0.010292	0.0007	14.711	3,011	2,450	54,498
Ajvide53	0.009491	0.001117	8.5	920	764	16,417
Ajvide58	0.009906	0.000441	22.476	29,847	24,550	534,726
Ajvide70	0.01018	0.00057	17.861	5,297	4,330	95,022
Ire8	0.009695	0.000916	10.588	1,315	1,082	23,981
Gökhem2	0.000697	0.000428	1.629	21,220	20,929	418,556
Gökhem4	0.001914	0.000943	2.029	1,078	1,038	20,804
Gökhem5	0.001455	0.001157	1.258	704	685	13,614
Gökhem7	0.00348	0.001569	2.218	378	351	7,621
Stuttgart	-0.004073	0.000368	-11.065	26,734	29,024	562,246

Supplementary Table 6. Admixture proportions based on *qpAdm*[35] analysis, specifying western hunter-gatherers (WHG), eastern hunter-gatherers (EHG), and Neolithic farmers (Barcın) as ancestral source populations.

test population	reference population	admixture proportion	n SNPs	chi square	tail prob
	WHG	1.000			
Bichon (LP)	EHG	0.000	374,266	3.52	0.74
	Barcın	0.000			
	WHG	1.000			
Rochedane (LP)	EHG	0.000	113, 744	6.72	0.35
	Barcın	0.000			
	WHG	1.000			
La Braña (M)	EHG	0.000	538,715	7.15	0.31
	Barcın	0.000			
	WHG	1.000			
Loschbour (M)	EHG	0.000	544,933	9.79	0.13
	Barcın	0.000			
	WHG	1.000			
Ranchot (M)	EHG	0.000	200,185	4.02	0.67
	Barcın	0.000			
	WHG	1.000			
Syltholm	EHG	0.000	115,800	6.34	0.39
	Barcın	0.000			
	WHG	0.000			
Karelia (M)	EHG	1.000	294,370	11.15	0.08
	Barcın	0.000			
	WHG	0.000			
Samara (M)	EHG	0.100	294,370	11.15	0.08
	NF	0.000			
	Barcın	0.441			
NorwayHG (M)	EHG	0.559	558,124	3.10	0.68
	Barcın	0.000			
	WHG	0.697			
Latvia (M)	EHG	0.303	560,151	4.49	0.48
	Barcın	0.000			
	WHG	0.649			
BalticHG (M)	EHG	0.351	562,935	3.46	0.63
	Barcın	0.000			
	WHG	0.593			
Motala (M)	EHG	0.407	545,689	4.83	0.44
	Barcın	0.000			
	WHG	0.780			
PWC (EN)	EHG	0.220	523,969	3.14	0.68
	Barcın	0.000			
	WHG	0.175			
Gökhem (EN)	EHG	0.000	407,865	2.68	0.75
	Barcın	0.825			
	WHG	0.180			
Iberia (EN)	EHG	0.000	557,569	2.98	0.70
	Barcın	0.820			
	WHG	0.162			
LBK (EN)	EHG	0.000	563,150	2.61	0.76
	Barcın	0.838			
	WHG	0.293			
GAC (EN)	EHG	0.000	563,197	9.72	0.08
	Barcın	0.707			

Supplementary Table 7. *D*-statistics of the form *D*(Yoruba, EHG; *X*, WHG) testing whether "*X*" forms a clade with WHG to the exclusion of EHG.

**Pop3 (*X*)**	***D*-stat**	**SE**	**Z**	**BABA**	**ABBA**	**SNPs**
Sylthom	-0.0173	0.007118	-2.432	4,736	4,903	115,687
La Braña	0.006	0.005081	1.176	23,498	23,219	538,716
Motala1	-0.0461	0.005356	-8.611	15,456	16,950	355,954
Motala2	-0.0475	0.005144	-9.232	18,888	20,771	441,690
Motala3	-0.0396	0.005355	-7.387	11,481	12,427	267,396
Motala4	-0.043	0.005249	-8.19	19,228	20,955	443,456
Motala6	-0.0522	0.005122	-10.192	16,280	18,073	380,891
Motala12	-0.0466	0.004941	-9.427	21,868	24,005	506,761
SBj	-0.0387	0.006137	-6.308	7,622	8,236	174,952
SF9	-0.0315	0.005791	-5.44	12,895	13,734	293,510
SF11	0.0071	0.007857	0.91	3,261	3,214	69,375
SF12	-0.0513	0.005394	-9.516	24,421	27,064	561,611
Hum1	-0.0443	0.006341	-6.99	9,391	10,262	207,167
Hum2	-0.0538	0.005228	-10.299	23,924	26,646	536,119
Steigen	-0.0605	0.00524	-11.544	18,646	21,047	421,170

Supplementary Table 8. *D*-statistics of the form *D*(Yoruba, Barcın; *X*, WHG) testing whether "*X*" forms a clade with WHG to the exclusion of Neolithic farmers (represented by Barcın).

Pop3 (*X*)	*D*-stat	SE	Z	BABA	ABBA	SNPs
Syltholm	0.0013	0.004313	0.307	4,954	4,941	121,065
La Braña	0.0054	0.003145	1.716	24,118	23,859	565,167
Ajvide52	-0.0008	0.005938	-0.128	2,447	2,450	54,498
Ajvide53	-0.016	0.009548	-1.673	740	764	16,417
Ajvide58	-0.0066	0.003166	-2.08	24,229	24,550	534,726
Ajvide70	-0.0066	0.004872	-1.361	4,273	4,330	95,022
Ire8	-0.0032	0.00813	-0.395	1,075	1082	23,981
Gökhem2	-0.043	0.003261	-13.183	19,203	20,929	418,556
Gökhem4	-0.0474	0.008241	-5.749	944	1,038	20,804
Gökhem5	-0.04	0.010509	-3.809	632	685	13,614
Gökhem7	-0.0341	0.012892	-2.648	328	351	7,621
Motala1	0.0005	0.003661	0.145	16,027	16,010	361,017
Motala2	-0.0006	0.00322	-0.19	19,676	19,700	449,904
Motala3	-0.005	0.003485	-1.445	11,647	11,765	268,527
Motala4	-0.0009	0.003462	-0.255	19,793	19,828	449,704
Motala6	-0.0026	0.003151	-0.833	16,841	16,930	384,398
Motala12	-0.0003	0.003127	-0.093	22,912	22,926	519,374
Stuttgart	-0.0506	0.003118	-16.231	26,228	29,024	562,246

References

1. Mortensen, M. F. *et al.* Fortidens spor og fremtidens forbindelse - bevaring og naturvidenskab på Femern Bælt projektet, Danmarks største arkæologiske udgravning. *Nationalmuseets Arbejdsmark* 22–36 (2015).
2. Groß, D. *et al.* People, lakes and seashores: Studies from the Baltic Sea basin and adjacent areas in the early and Mid-Holocene. *Quat. Sci. Rev.* **185**, 27–40 (2018).
3. Jensen, L. E. *et al.* Syltholmudgravningerne - jagten på stenalderens jægere, fiskere og bønder i et druknet landskab. *Aarbøger for nordisk Oldkyndighed og Historie* (Copenhagen, 2018).
4. Sørensen, S. A. Danmarks største stenalderudgravning – Fantastiske fund fra Femernudgravningerne. *FUND & FORTID* **2**, 17–24 (2018).
5. Sørensen, S. A. Syltholm: Denmark's largest Stone Age excavation. *Mesolithic Miscellany* **24**, 3–10 (2016).
6. Brock, F., Higham, T., Ditchfield, P. & Ramsey, C. B. Current Pretreatment Methods for AMS Radiocarbon Dating at the Oxford Radiocarbon Accelerator Unit (Orau). *Radiocarbon* **52**, 103–112 (2010).
7. Ramsey, C. B. Radiocarbon Calibration and Analysis of Stratigraphy: The OxCal Program. *Radiocarbon* **37**, 425–430 (1995).
8. Mills, J. J. S. & White, R. *The Organic Chemistry of Museum Objects*. (Butterworth-Heinemann, 1987).
9. Allentoft, M. E. *et al.* The half-life of DNA in bone: measuring decay kinetics in 158 dated fossils. *Proc Biol Sci. 279(1748):4724-33* (2012).
10. Deagle, B. E., Eveson, J. P. & Jarman, S. N. Quantification of damage in DNA recovered from highly degraded samples-a case study on DNA in faeces. *Front. Zool.* **3**, 11 (2006).
11. Olalde, I. *et al.* Derived immune and ancestral pigmentation alleles in a 7,000-year-old Mesolithic European. *Nature* **507**, 225–228 (2014).
12. Schroeder, H. *et al.* Origins and genetic legacies of the Caribbean Taino. *Proc. Natl. Acad. Sci. U. S. A.* **22**, 201716839–201716836 (2018).
13. Warinner, C. *et al.* A Robust Framework for Microbial Archaeology. *Annu. Rev. Genomics Hum. Genet.* **18**, 321–356 (2017).
14. Kryukov, K. & Imanishi, T. Human Contamination in Public Genome Assemblies. *PLoS One* **11**, e0162424 (2016).
15. Coghlan, A., Gordon, D. & Berriman, M. Contamination screening of parasitic worm genome assemblies. Preprint at https://protocolexchange.researchsquare.com/article/nprot-6669/v1.
16. Pedersen, M. W. *et al.* Postglacial viability and colonization in North America's ice-free corridor. *Nature* **537**, 45–49 (2016).
17. Vågene, Å. J. *et al.* Salmonella enterica genomes from victims of a major sixteenth-century epidemic in Mexico. *Nat Ecol Evol* **2**, 520–528 (2018).
18. Key, F. M., Posth, C., Krause, J., Herbig, A. & Bos, K. I. Mining Metagenomic Data Sets for Ancient DNA: Recommended Protocols for Authentication. *Trends Genet.* **33**, 508–520 (2017).
19. Huebler, R., Key, F. M. M., Warinner, C., Bos, K. I. & Krause, J. HOPS: Automated

detection and authentication of pathogen DNA in archaeological remains. Preprint at https://www.biorxiv.org/content/10.1101/534198v2 (2018).
20. Truong, D. T. *et al.* MetaPhlAn2 for enhanced metagenomic taxonomic profiling. *Nat. Methods* **12**, 902–903 (2015).
21. Li, H. & Durbin, R. Fast and accurate short read alignment with Burrows-Wheeler transform. *Bioinformatics* **25**, 1754–1760 (2009).
22. Picard Tools - By Broad Institute. Available at: http://broadinstitute.github.io/picard/. (Accessed: 15th December 2018).
23. Jónsson, H., Ginolhac, A., Schubert, M., Johnson, P. L. F. & Orlando, L. mapDamage2.0: fast approximate Bayesian estimates of ancient DNA damage parameters. *Bioinformatics* **29**, 1682–1684 (2013).
24. Quinlan, A. R. & Hall, I. M. BEDTools: a flexible suite of utilities for comparing genomic features. *Bioinformatics* **26**, 841–842 (2010).
25. Krzywinski, M. *et al.* Circos: an information aesthetic for comparative genomics. *Genome Res.* **19**, 1639–1645 (2009).
26. Skoglund, P. *et al.* Separating endogenous ancient DNA from modern day contamination in a Siberian Neandertal. *Proc. Natl. Acad. Sci. U. S. A.* **111**, 2229–2234 (2014).
27. Team, R. C. R: A language and environment for statistical computing. Vienna: R Foundation for Statistical Computing (2014).
28. Thorvaldsdóttir, H., Robinson, J. T. & Mesirov, J. P. Integrative Genomics Viewer (IGV): high-performance genomics data visualization and exploration. *Brief. Bioinform.* **14**, 178–192 (2013).
29. Lu, J. & Salzberg, S. L. Removing contaminants from databases of draft genomes. *PLoS Comput. Biol.* **14**, e1006277 (2018).
30. Astrup, P. M. *Sea-level change in Mesolithic southern Scandinavia. Long- and short-term effects on society and the environment*. Jysk Arkæologisk Selskabs Skrifter **106** (Jutland Archaeological Society, 2018).
31. The Human Microbiome Project Consortium *et al.* Structure, function and diversity of the healthy human microbiome. *Nature* **486**, 207-214 (2012).
32. Rasmussen, M. *et al.* The genome of a Late Pleistocene human from a Clovis burial site in western Montana. *Nature* **506**, 225–229 (2014).
33. Rasmussen, M. *et al.* The ancestry and affiliations of Kennewick Man. *Nature* **523**, 455–458 (2015).
34. Renaud, G., Slon, V., Duggan, A. T. & Kelso, J. Schmutzi: estimation of contamination and endogenous mitochondrial consensus calling for ancient DNA. *Genome Biol.* **16**, 224 (2015).
35. Haak, W. *et al.* Massive migration from the steppe was a source for Indo-European languages in Europe. *Nature* **522**, 207-211 (2015).
