# Supplementary material for: A 5700 year-old human genome and oral microbiome from chewed birch pitch
Source: Nat Commun. 2019 Dec 17;10:5520. doi: 10.1038/s41467-019-13549-9 (PMC6917805; doi:10.1038/s41467-019-13549-9)
Supplement: Supplementary file 2 — Description of Additional Supplementary Files [file 41467_2019_13549_MOESM2_ESM.pdf]

Title: Supplementary Dataset 1.
Description: Imputed genotype likelihoods

Title: Supplementary Dataset 2.
Description: HIrisPlex-S results

Title: Supplementary Dataset 3.
Description: List of previously published ancient genomes used for population genetics analyses

Title: Supplementary Dataset 4.
Description: MetaPhlan2 results

Title: Supplementary Dataset 5.
Description: MALT results

Title: Supplementary Dataset 6.
Description: Pneumococcal virulence factors

Title: Supplementary Dataset 7.
Description: HOLI results
