# Supplementary material for: A 5700 year-old human genome and oral microbiome from chewed birch pitch
Source: Nat Commun. 2019 Dec 17;10:5520. doi: 10.1038/s41467-019-13549-9 (PMC6917805; doi:10.1038/s41467-019-13549-9)
Supplement: Supplementary file 3 — Reporting Summary [file 41467_2019_13549_MOESM3_ESM.pdf]

nature research

Corresponding author(s): Hannes Schroeder

Last updated by author(s): Nov 13, 2019

Reporting Summary

Nature Research wishes to improve the reproducibility of the work that we publish. This form provides structure for consistency and transparency in reporting. For further information on Nature Research policies, see Authors & Referees and the Editorial Policy Checklist.

Statistics

For all statistical analyses, confirm that the following items are present in the figure legend, table legend, main text, or Methods section.

n/a	Confirmed	
☐	☒	The exact sample size (*n*) for each experimental group/condition, given as a discrete number and unit of measurement
☒	☐	A statement on whether measurements were taken from distinct samples or whether the same sample was measured repeatedly
☐	☒	The statistical test(s) used AND whether they are one- or two-sided<br>*Only common tests should be described solely by name; describe more complex techniques in the Methods section.*
☒	☐	A description of all covariates tested
☐	☒	A description of any assumptions or corrections, such as tests of normality and adjustment for multiple comparisons
☐	☒	A full description of the statistical parameters including central tendency (e.g. means) or other basic estimates (e.g. regression coefficient) AND variation (e.g. standard deviation) or associated estimates of uncertainty (e.g. confidence intervals)
☒	☐	For null hypothesis testing, the test statistic (e.g. *F*, *t*, *r*) with confidence intervals, effect sizes, degrees of freedom and *P* value noted<br>*Give P values as exact values whenever suitable.*
☒	☐	For Bayesian analysis, information on the choice of priors and Markov chain Monte Carlo settings
☒	☐	For hierarchical and complex designs, identification of the appropriate level for tests and full reporting of outcomes
☒	☐	Estimates of effect sizes (e.g. Cohen's *d*, Pearson's *r*), indicating how they were calculated

Our web collection on statistics for biologists contains articles on many of the points above.

Software and code

Policy information about availability of computer code

Data collection	n/a
Data analysis	Base calling was done using Illumina's bcl2fastq2 conversion software v2.20.0. FastQ files were processed using PALEOMIX v1.2.12. Adapters and low quality reads (Q<20) were removed using AdapterRemoval v2.2.0. Trimmed and filtered reads were then mapped to hg19 (build 37.1) using BWA. MapDamage 2.0.954 was used to evaluate the authenticity of the retained reads. Schmutzi was used to determine the endogenous consensus mtDNA sequences and to estimate present-day human contamination. The mtDNA haplogroup was determined using Haplogrep v2.2. Genotype likelihoods were computed using ANGSD. Missing genotypes were imputed using impute2. Principal component analysis was performed using smartPCA. D- and f-statistics were computed using AdmixTools. Admixture proportions were modeled using qpAdm. The non-human reads were analysed using MetaPhlan2, MALT and the Holi pipeline. Coverage statistics of the individual alignments were obtained using Bedtools and plotted in Circos. Mappability was estimated using GEM2. Contigs were assembled using megahit. Contigs were aligned against the Virulence Factor Database (downloaded 22/11-2018) using BLASTn. Radiocarbon dates were calibrated using OxCal v 4.3.

For manuscripts utilizing custom algorithms or software that are central to the research but not yet described in published literature, software must be made available to editors/reviewers. We strongly encourage code deposition in a community repository (e.g. GitHub). See the Nature Research guidelines for submitting code & software for further information.

Data

Policy information about availability of data

All manuscripts must include a data availability statement. This statement should provide the following information, where applicable:
- Accession codes, unique identifiers, or web links for publicly available datasets
- A list of figures that have associated raw data
- A description of any restrictions on data availability

The ancient reads are available for download at the European Nucleotide Archive under accession number PRJEB30280.

Field-specific reporting

Please select the one below that is the best fit for your research. If you are not sure, read the appropriate sections before making your selection.

☒ Life sciences ☐ Behavioural & social sciences ☐ Ecological, evolutionary & environmental sciences

For a reference copy of the document with all sections, see nature.com/documents/nr-reporting-summary-flat.pdf

Life sciences study design

All studies must disclose on these points even when the disclosure is negative.

Sample size	1 plus 100s of samples for comparison.
Data exclusions	Delftia spp. were excluded from the metagenomic analysis as known laboratory contaminants.
Replication	n/a
Randomization	n/a
Blinding	n/a

Reporting for specific materials, systems and methods

We require information from authors about some types of materials, experimental systems and methods used in many studies. Here, indicate whether each material, system or method listed is relevant to your study. If you are not sure if a list item applies to your research, read the appropriate section before selecting a response.

Materials & experimental systems

n/a	Involved in the study	
☒	☐	Antibodies
☒	☐	Eukaryotic cell lines
☐	☒	Palaeontology
☒	☐	Animals and other organisms
☒	☐	Human research participants
☒	☐	Clinical data

Methods

n/a	Involved in the study	
☒	☐	ChIP-seq
☒	☐	Flow cytometry
☒	☐	MRI-based neuroimaging

Palaeontology

Specimen provenance	The sample was excavated at the site of Syltholm on the island of Lolland in Denmark and was obtained from the Museum Lolland-Falster.
Specimen deposition	The specimen is being stored at the Museum Lolland-Falster.
Dating methods	The specimen was directly dated at the Centre for Isotope Research at the University of Groningen (GrM-13305). The dates were calibrated using OxCal v 4.3 and the IntCal13 calibration curve.

☒ Tick this box to confirm that the raw and calibrated dates are available in the paper or in Supplementary Information.
